# Protein farnesylation: from molecular mechanisms to therapeutic targeting in cancer, aging, and beyond

**DOI:** 10.3389/fphar.2026.1907367

**Published:** 2026-08-14

**Authors:** Jiacheng Lou, Danlu Zhang, Yingjie Wang

**Affiliations:** 1 Department of Pediatrics, The Second Hospital of Dalian Medical University, Dalian, China; 2 Human Resources Department, The Second Hospital of Dalian Medical University, Dalian, China; 3 Pediatric Oncology and Hematology Center, The Second Affiliated Hospital of Dalian Medical University, Dalian, China

**Keywords:** farnesylation, farnesyltransferase inhibitors, post-translational modification, protein prenylation, Ras GTPases

## Abstract

Protein farnesylation, catalyzed by farnesyltransferase (FTase), covalently attaches a 15-carbon farnesyl lipid to cysteine residues within C-terminal CaaX motifs. This modification governs the subcellular localization, protein–protein interactions, and activity of diverse eukaryotic proteins, including Ras GTPases, nuclear lamins, molecular chaperones, and kinetochore components. This review examines FTase enzymology, substrate recognition, and the sequential post-farnesylation processing by RCE1 and ICMT, alongside the expanding farnesylated proteome and its roles in signaling, nuclear integrity, and mitosis. Central to oncogenic Ras function, farnesylation drove the development of FTase inhibitors (FTIs) as anticancer agents; although alternative prenylation of K-Ras and N-Ras limits efficacy in solid tumors, FTIs show clinical activity in hematologic malignancies. The critical importance of farnesylation is highlighted in Hutchinson–Gilford progeria syndrome, where permanently farnesylated progerin disrupts nuclear architecture, and the FTI lonafarnib extends patient survival. Emerging roles—including driving K-Ras liquid–liquid phase separation and contributing to neurodegeneration and pulmonary hypertension—further broaden the pathophysiological relevance of this modification. Targeting farnesylation through dual prenylation inhibitors, PROTACs, and mevalonate pathway modulation offers promising therapeutic strategies across cancer, progeroid syndromes, and age-related diseases.

## Introduction

Protein prenylation is a fundamental post-translational modification that involves the covalent attachment of isoprenoid lipids—either a 15-carbon farnesyl or a 20-carbon geranylgeranyl moiety—to cysteine residues at or near the carboxyl terminus of target proteins. This lipidation is essential for the proper subcellular localization, protein–protein interactions, and biological activity of a diverse array of eukaryotic proteins, including members of the Ras superfamily of small GTPases, nuclear lamins, heterotrimeric G-protein subunits, and molecular chaperones (Casey et al., 1989; Manne et al., 1990; Reiss et al., 1990). The discovery that oncogenic Ras proteins require farnesylation for membrane association and transforming activity provided the initial impetus for understanding this modification (Casey et al., 1989; Kato et al., 1992). Seminal work in the late 1980s and early 1990s demonstrated that the conserved C-terminal CaaX motif—where C is cysteine, A is an aliphatic amino acid, and X is any amino acid—directs farnesylation of the cysteine residue, followed by proteolytic removal of the -aaX tripeptide and carboxyl methylation of the newly exposed prenylcysteine (Hancock et al., 1991; Perez-Sala et al., 1991; Ashby et al., 1992). These sequential processing steps are critical for the efficient membrane binding and function of CaaX proteins (Hancock et al., 1991; Kuroda et al., 1993).

The enzyme responsible for catalyzing the transfer of the farnesyl group from farnesyl diphosphate (FPP) to the cysteine of the CaaX motif is protein farnesyltransferase (FTase). FTase was first purified and characterized from rat brain cytosol as a heterodimeric zinc metalloenzyme composed of α- and β-subunits (Reiss et al., 1990; He et al., 1991). The α-subunit is shared with the related enzyme protein geranylgeranyltransferase type I (GGTase-I), while the β-subunit confers substrate specificity for the CaaX sequence (Finegold et al., 1991; Yokoyama et al., 1991). The identity of the X residue largely determines whether a protein is farnesylated or geranylgeranylated: when X is methionine, serine, alanine, or glutamine, the protein is recognized by FTase, whereas a leucine or isoleucine at the X position directs geranylgeranylation by GGTase-I (Finegold et al., 1991; Kinsella et al., 1991; Yokoyama et al., 1991). Structural studies have revealed the molecular basis for CaaX peptide recognition and the catalytic mechanism of FTase, including the processive nature of the reaction and the basis for inhibition by peptidomimetics (Long et al., 2001; 2002; Yang et al., 2012).

The biological significance of protein farnesylation extends across virtually all aspects of eukaryotic cell biology. Farnesylation is essential for the membrane targeting and signaling functions of Ras proteins, which act as molecular switches controlling cell proliferation, differentiation, and survival (Aronheim et al., 1994; Stokoe et al., 1994; Prior et al., 2001; Pylayeva-Gupta et al., 2011). Beyond Ras, farnesylation regulates the assembly and dynamics of the nuclear lamina through modification of prelamin A and lamin B, which are critical for nuclear architecture and chromatin organization (Lutz et al., 1992; Firmbach-Kraft and Stick, 1995; Maske et al., 2003; Vergnes et al., 2004). In the visual system, farnesylation of the transducin γ-subunit is required for light signaling and light-dependent translocation in photoreceptor cells (Ohguro et al., 1991; Kassai et al., 2005). Farnesylation also modulates the function of molecular chaperones such as Hsp40 proteins in both plants and animals (Zhu et al., 1993; Barghetti et al., 2017), SNARE proteins involved in membrane fusion (Fukasawa et al., 2004; Wen et al., 2010), and mitotic checkpoint proteins that ensure faithful chromosome segregation (Moudgil et al., 2015; Mosalaganti et al., 2017; Sacristan et al., 2018; Wu et al., 2024). In plants, farnesylation plays critical roles in meristem development, hormone signaling, and stress responses (Yalovsky et al., 2000b; 2000a; Ziegelhoffer et al., 2000; Northey et al., 2016; Wu et al., 2017; Feng et al., 2021).

Given its central role in the function of oncogenic Ras proteins, FTase emerged as a compelling therapeutic target for cancer (Berndt et al., 2011). The development of FTase inhibitors (FTIs) was spurred by the demonstration that CaaX tetrapeptides could competitively inhibit FTase *in vitro* (Reiss et al., 1990; Brown et al., 1992), leading to the design of potent peptidomimetic inhibitors such as the benzodiazepine-based compounds (James et al., 1993) and the CaaX mimetic FTI-276 (Sun et al., 1995). These inhibitors were shown to selectively block Ras farnesylation, reverse the transformed phenotype of Ras-driven tumor cells, and inhibit tumor growth in preclinical models (Kohl et al., 1993; Nagasu et al., 1995; Sepp-Lorenzino et al., 1995; Sun et al., 1995). Importantly, FTIs also demonstrated activity against tumor cells lacking Ras mutations, indicating that other farnesylated proteins contribute to their anti-tumor effects (Sepp-Lorenzino et al., 1995; End et al., 2001). Clinical development of FTIs, including tipifarnib (R115777), lonafarnib (SCH66336), and BMS-214662, has been pursued across a range of hematologic malignancies and solid tumors (Adjei et al., 2000; Karp et al., 2001; Cohen et al., 2003; Alsina et al., 2004; Zimmerman et al., 2004; Lancet et al., 2007; Feldman et al., 2008).

Beyond oncology, the importance of protein farnesylation is dramatically illustrated by the premature aging disorder Hutchinson-Gilford progeria syndrome (HGPS). HGPS is caused by a mutant form of prelamin A, termed progerin, that retains a farnesyl lipid anchor due to a deletion that removes the ZMPSTE24 cleavage site required for its removal (Capell et al., 2005; Mallampalli et al., 2005; Yang et al., 2005). The accumulation of permanently farnesylated progerin at the nuclear envelope disrupts nuclear architecture, leading to the characteristic nuclear blebbing and cellular senescence observed in HGPS cells (Capell et al., 2005; Toth et al., 2005; Cao et al., 2007; Dechat et al., 2007). FTIs have been shown to improve nuclear morphology in HGPS fibroblasts and extend survival in mouse models and human clinical trials, leading to the FDA approval of lonafarnib (Zokinvy) for the treatment of HGPS (Toth et al., 2005; Yang et al., 2005; Gordon et al., 2014; 2016; Misteli, 2021). This therapeutic success has further highlighted the broad pathophysiological relevance of protein farnesylation.

This review provides a comprehensive overview of protein farnesylation, encompassing the molecular mechanisms of FTase enzymology and CaaX processing, the expanding repertoire of farnesylated proteins and their cellular functions, the role of farnesylation in human diseases including cancer and progeroid syndromes, and the development of FTIs as therapeutic agents. We will discuss the lessons learned from preclinical models and clinical trials, the challenges of resistance and alternative prenylation, and emerging strategies to target farnesylation for therapeutic benefit.

### Molecular enzymology of farnesyltransferase

**FIGURE 1:**
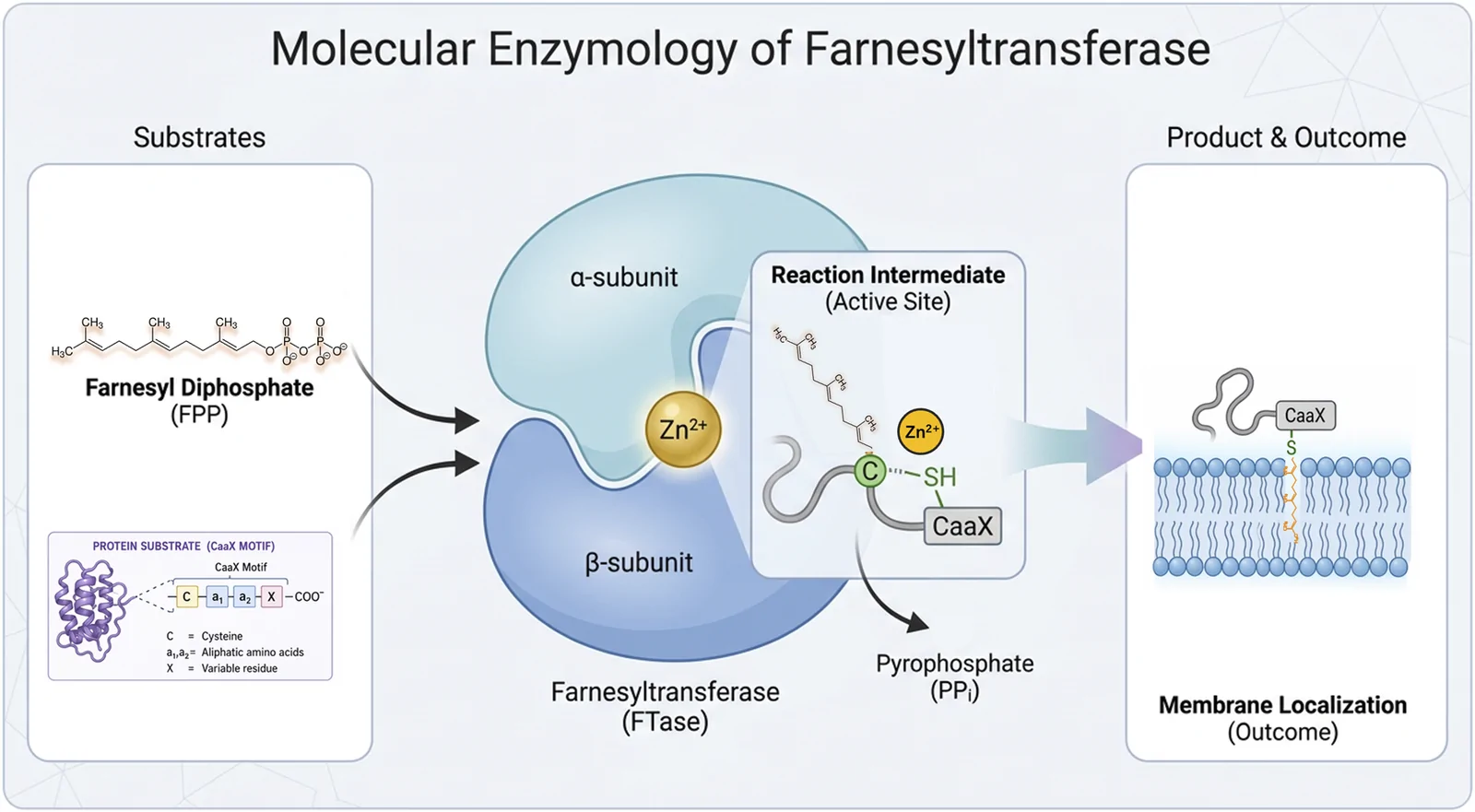
Molecular enzymology and catalytic mechanism of Farnesyltransferase (FTase) (Left) Substrate Recognition: The farnesylation reaction requires two primary substrates: Farnesyl Diphosphate (FPP), which acts as the 15-carbon isoprenoid lipid donor, and a target protein substrate featuring a consensus C-terminal CaaX motif (where ‘C’ represents the target cysteine, ‘a’ denotes an aliphatic amino acid, and ‘X’ is a variable residue dictating enzyme specificity) (Center) Catalytic Mechanism: FTase is a heterodimer composed of an α-subunit and a β-subunit. At the enzyme’s active site, a strictly conserved catalytic zinc ion (Zn^2+^) coordinates the sulfhydryl group (-SH) of the CaaX cysteine, lowering its pKa and activating it as a nucleophile. The subsequent transfer reaction involves the nucleophilic attack of the cysteine thiolate on the C1 carbon of FPP (Right) Product Formation and Functional Outcome: This reaction covalently links the farnesyl group to the target protein via a stable thioether bond, accompanied by the displacement and release of inorganic pyrophosphate (PP_i_). The resulting farnesylated protein possesses a hydrophobic lipid tail, which acts as a critical molecular determinant for its subsequent functional outcome—directing the stable anchoring and localization of the protein to cellular membranes.

Protein farnesyltransferase (FTase) is a heterodimeric zinc metalloenzyme that catalyzes the transfer of a 15-carbon farnesyl isoprenoid from farnesyl diphosphate (FPP) to the cysteine residue of protein substrates bearing a C-terminal CaaX motif, where C is cysteine, ‘a’ represents aliphatic amino acids, and X is a variable residue that influences isoprenoid specificity (Reiss et al., 1990; Kinsella et al., 1991) (Figure 1) (Table 1). The enzyme is composed of an α-subunit and a β-subunit; the α-subunit is shared with the related enzyme geranylgeranyltransferase type I (GGTase-I), while the β-subunit dictates substrate specificity (Goodman et al., 1990; He et al., 1991). The catalytic mechanism is zinc-dependent, with the active site zinc ion coordinating the cysteine thiol of the CaaX substrate to facilitate nucleophilic attack on the C1 carbon of FPP (Long et al., 2002). Detailed structural studies have captured a complete series of snapshots along the reaction coordinate, revealing an unusual processive mechanism in which product release is coupled to substrate binding (Long et al., 2002). This processivity is analogous to that observed in the homologous Rab geranylgeranyltransferase, suggesting a structural basis for the sequential addition of multiple prenyl groups (Long et al., 2002).

**TABLE 1:** Key findings in molecular enzymology of farnesyltransferase.

Name	Function	Citation
FTase	Heterodimeric zinc metalloenzyme; transfers farnesyl from FPP to CaaX cysteine	Reiss et al. (1990), Kinsella et al. (1991)
α-subunit	Shared with GGTase-I	Goodman et al. (1990), He et al. (1991)
β-subunit	Dictates substrate specificity	Goodman et al. (1990), He et al. (1991)
Catalytic zinc	Coordinates CaaX cysteine thiol for nucleophilic attack on FPP	Long et al. (2002)
Processive mechanism	Product release coupled to substrate binding; analogous to Rab GGTase	Long et al. (2002)
α-secondary ^3^H KIE (fast substrate)	Value near unity; indicates conformational change is rate-limiting	Pais et al. (2006)
CVIM transition state	Dissociative S_n_2-like character; substantial C–O bond breaking before C–S bond formation	Yang et al. (2012)
CVLS transition state	Symmetric S_n_2; equal bond making/breaking at trigonal bipyramidal carbon	Yang et al. (2012)
CaaX X-position specificity	Met/Ser directs farnesylation; Leu/Ile directs geranylgeranylation	Finegold et al. (1991), Kinsella et al. (1991)
CaaX binding conformation	Extended conformation in β-subunit hydrophobic pocket; X residue in specificity pocket	Long et al. (2001)
CVFM peptide	Aromatic at A2; pure inhibitor; resistance overcome by N-terminal acylation/extension	Long et al. (2001)

Kinetic isotope effect (KIE) studies have provided critical insight into the transition state architecture of FTase. Measurement of the α-secondary ^3^H KIE at the C1 position of FPP yielded a value near unity for a rapidly farnesylated peptide substrate, indicating that a conformational change, rather than the chemical step of farnesylation, is rate-limiting for this substrate (Pais et al., 2006). However, the magnitude of the KIE is substrate-dependent; computational studies using quantum mechanical/molecular mechanical (QM/MM) methods have demonstrated that the transition state for the CVIM peptide substrate exhibits dissociative S_n_2-like character, whereas the CVLS substrate proceeds through a symmetric S_n_2 transition state, consistent with the absence of a measurable α-secondary KIE for the latter (Yang et al., 2012). These QM/MM potential of mean force calculations coupled with umbrella sampling techniques further revealed that the experimentally observed transition state for CVIM involves substantial bond breaking between the C1 carbon of FPP and the diphosphate leaving group prior to significant bond formation with the cysteine thiolate nucleophile, while the symmetric S_n_2 transition state for CVLS features nearly equal degrees of bond making and bond breaking at the trigonal bipyramidal carbon center (Yang et al., 2012). These findings highlight a mechanistic dichotomy in which the enzyme can accommodate subtle alterations in transition state structure depending on the peptide substrate, demonstrating facile substrate-dependent modulation of the chemical landscape catalyzed by FTase (Yang et al., 2012).

Substrate recognition by FTase is primarily governed by the CaaX tetrapeptide. The enzyme exhibits a strong preference for a C-terminal methionine or serine at the X position for farnesylation, whereas a leucine or isoleucine directs geranylgeranylation by GGTase-I (Finegold et al., 1991; Kinsella et al., 1991). The structural basis for this specificity has been elucidated through crystallographic studies of FTase complexed with various CaaX peptides and peptidomimetic inhibitors (Long et al., 2001). These structures reveal that the CaaX peptide binds in an extended conformation within a hydrophobic pocket of the β-subunit, with the cysteine thiol positioned for zinc coordination and the X residue accommodated in a specificity pocket that discriminates between farnesyl and geranylgeranyl transfer (Long et al., 2001). Notably, tetrapeptides containing an aromatic residue at the A2 position, such as CVFM, act as pure inhibitors because they bind to the enzyme but cannot undergo farnesylation; this resistance is overcome by N-terminal acylation or extension, which neutralizes the positive charge on the cysteine amino group (Brown et al., 1992). The development of benzodiazepine-based peptidomimetics that mimic the peptide turn of the CaaX motif has yielded potent FTase inhibitors with IC_50_ values below 1 nM, which effectively block Ras farnesylation in cells and reverse the transformed phenotype (James et al., 1993).

The comparison between FTase and GGTase-I highlights key determinants of isoprenoid specificity. While both enzymes recognize CaaX motifs, GGTase-I preferentially modifies substrates with a C-terminal leucine (Finegold et al., 1991). This specificity is not absolute, as cross-prenylation can occur under certain conditions. For instance, K-Ras, which terminates with a methionine, is normally farnesylated but can be geranylgeranylated by GGTase-I when FTase is inhibited, providing a mechanism of resistance to farnesyltransferase inhibitors (FTIs) (James et al., 1996; Lobell et al., 2001). Similarly, RhoB, which terminates with a CKVL motif, is uniquely both farnesylated and geranylgeranylated *in vivo*, with its prenylation determined by the three C-terminal amino acids (Baron et al., 2000). The biological consequences of differential isoprenylation are profound, as demonstrated by studies in yeast where mutation of the a-factor farnesylation motif to a geranylgeranylation target altered both its export and bioactivity (Caldwell et al., 1994). Recent chemical proteomic studies using dual alkyne-tagged isoprenoid probes (YnF and YnGG) have enabled quantitative, system-wide analysis of prenylation dynamics, revealing that RRAS2 is prenylated by both FTase and GGTase-I under physiological conditions, and that K-Ras, N-Ras, and RRAS2 exhibit robust dose-dependent increases in geranylgeranylation upon FTI treatment, confirming switch-like alternative prenylation behavior for these substrates (Storck et al., 2019).

The processive nature of FTase is further reflected in the sequential post-translational modifications that follow farnesylation. After prenylation, the C-terminal -aaX tripeptide is cleaved by the endoprotease RCE1, and the newly exposed farnesylcysteine is methylated by isoprenylcysteine carboxyl methyltransferase (ICMT) (Hancock et al., 1991; Ashby et al., 1992). These modifications are essential for proper membrane targeting and protein function, as demonstrated for lamin B1, where RCE1-dependent endoproteolysis and carboxymethylation are required for nuclear lamina organization (Maske et al., 2003). The farnesylation-dependent processing of prelamin A to mature lamin A involves an additional cleavage step by the zinc metalloprotease ZMPSTE24, which removes the terminal 15 amino acids including the farnesylated cysteine (Lutz et al., 1992). Defects in this processing pathway lead to the accumulation of farnesylated prelamin A and are associated with progeroid disorders (Ragnauth et al., 2010; Yang et al., 2024). The critical role of farnesylation in lamin biology is underscored by the finding that farnesylation of lamin B1 is essential for nuclear chromatin retention during neuronal migration, with nonfarnesylated lamin B1 leading to perinatal lethality and severe neurodevelopmental defects (Jung et al., 2013). In contrast, lamin B2 farnesylation is dispensable for normal development (Jung et al., 2013). The farnesylation of prelamin A also directs nuclear positioning in differentiating human myoblasts by recruiting SUN1 to the nuclear envelope (Mattioli et al., 2011). Furthermore, the permanently farnesylated lamin A mutant progerin, which causes Hutchinson-Gilford progeria syndrome, disrupts nuclear architecture and nucleocytoskeletal connections, effects that can be ameliorated by inhibiting protein farnesylation (Chang et al., 2019; Foo et al., 2024). Direct detection of prenylated and processed peptides at the whole proteome level has now been achieved using multifunctional capture reagents, confirming that YnF and YnGG labeling is compatible with endogenous post-prenylation processing by RCE1 and ICMT, and revealing novel C-terminal processing of geranylgeranylated RAB3B, RAB3D, and RAB7A in which the two terminal amino acids of the canonical XCXC motif are removed (Storck et al., 2019).

### The farnesylated proteome: key substrates and functional consequences

**FIGURE 2:**
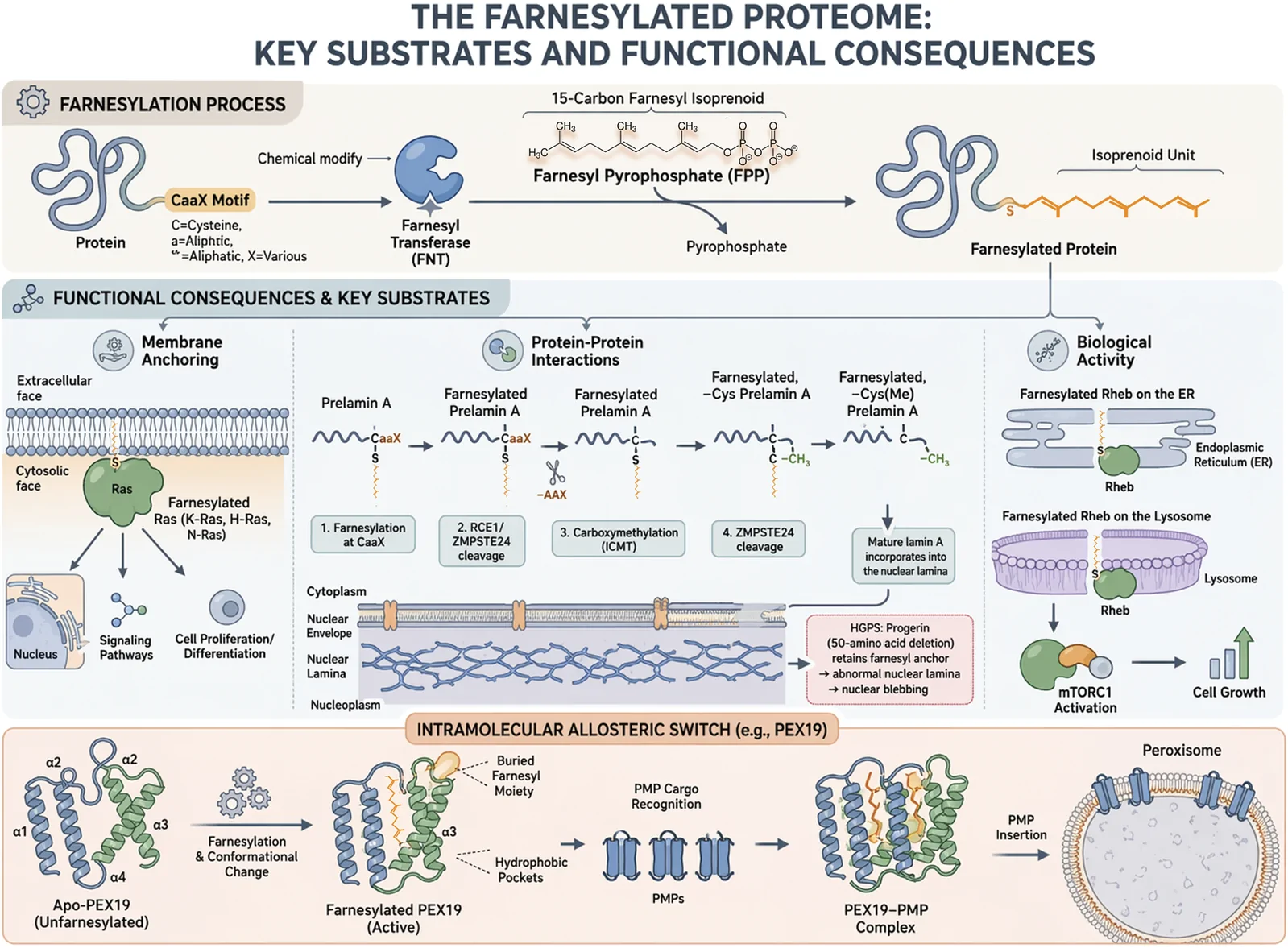
Overview of the farnesylated proteome and its functional consequences. farnesylation process (Top): Farnesyltransferase (FNT) catalyzes the covalent attachment of a 15-carbon farnesyl isoprenoid (from FPP) to target proteins containing a C-terminal “CaaX” motif. Functional Consequences (Middle): This lipid modification dictates key cellular functions through three main mechanisms:Membrane Anchoring: Anchoring proteins like Ras GTPases to the plasma membrane for essential signaling and cell proliferation. Protein-Protein Interactions: Facilitating complex assembly, such as Pre-lamin A in nuclear lamina formation. Biological Activity: Regulating distinct pathways, exemplified by RheB’s role in driving mTOR signaling for cell growth. Allosteric Switch Mechanism (Bottom): Beyond membrane anchoring, farnesylation can drive dramatic structural changes. In the chaperone PEX19, the attached farnesyl group is buried internally, triggering a conformational shift that creates a hydrophobic cavity. This active conformation is required to bind and import Peroxisomal Membrane Proteins (PMPs). Import.

Farnesylation—the post-translational addition of a 15-carbon farnesyl isoprenoid to a cysteine within a C-terminal CaaX motif—governs subcellular localization, protein-protein interactions, and biological activity of diverse proteins. Beyond membrane anchoring, farnesylation can act as an intramolecular allosteric switch, as demonstrated for the peroxisomal import receptor PEX19. Structural studies reveal the farnesyl moiety is buried within an internal hydrophobic cavity formed by helices α1–α4, inducing conformational changes that create two hydrophobic pockets for recognition of peroxisomal membrane protein (PMP) cargoes (Emmanouilidis et al., 2017). This allosteric mechanism enhances PMP binding affinity approximately seven-fold; mutations disrupting farnesyl contacts (e.g., M179R, I195K, M255R) abolish cargo binding and fail to complement the ΔPEX19 phenotype in Zellweger patient fibroblasts (Emmanouilidis et al., 2017) (Figure 2) (Table 2).

**TABLE 2:** Key farnesylated proteins and their functional roles.

Name	Function	Citation
PEX19	Farnesylation acts as an intramolecular allosteric switch, inducing conformational changes that create hydrophobic pockets for PMP cargo recognition; enhances binding affinity ∼7-fold	Emmanouilidis et al. (2017)
CPX III	Farnesylated complexin isoform at retinal ribbon and conventional amacrine synapses; farnesylation regulates synaptic targeting and transmitter release modulation	Reim et al. (2005)
CPX IV	Farnesylated complexin isoform selectively expressed at photoreceptor and bipolar cell ribbon synapses; enriched in rod terminals	Reim et al. (2005)
H-Ras	Farnesylation required for palmitoylation, plasma membrane trafficking, and downstream effector activation; FTI-sensitive	Reuveni et al. (2000)
K-Ras4B	Farnesylation and polybasic domain needed for membrane binding; carboxymethylation enhances interaction; drives liquid-liquid phase separation and oncogenic signaling	Wang et al. (2026)
Rheb	Farnesylation required for mTORC1 activation; FTI-sensitive	Mavrakis et al. (2008)

In the nervous system, farnesylation regulates synaptic transmission. The complexin (CPX) family includes CPX III and CPX IV, the only CPX isoforms at retinal ribbon synapses, which contain C-terminal CAAX-box motifs directing farnesylation (Reim et al., 2005). Unlike soluble CPXs I and II, CPX III associates with cytosolic and membrane fractions, including synaptic membranes and the crude synaptic vesicular fraction, and its COOH-terminal farnesylation regulates synaptic targeting and modulatory function in transmitter release (Reim et al., 2005). At retinal ribbon synapses, where sensory neurons sustain exocytosis rates of several hundred vesicles per second per release site, CPX IV is selectively expressed at photoreceptor and bipolar cell ribbon synapses, while CPX III localizes to both ribbon and conventional amacrine cell synapses (Reim et al., 2005). Their differential distribution—CPX IV enriched in rod terminals, CPX III predominantly in cone terminals—suggests farnesylation-dependent membrane targeting contributes to the unique release efficacy and functional specialization of retinal sensory neurons (Reim et al., 2005).

### Ras superfamily GTPases: membrane targeting and oncogenic signaling

The Ras superfamily of small GTPases represents the most extensively studied class of farnesylated proteins, where the lipid anchor is essential for membrane association and biological activity. The three canonical Ras isoforms—H-Ras, N-Ras, and K-Ras4B—all require farnesylation for proper function. For H-Ras, farnesylation is a prerequisite for subsequent palmitoylation and trafficking to the plasma membrane to activate downstream effectors like Prior et al. (2001). A farnesylated but non-proteolyzed and non-methylated K-Ras mutant still exhibits approximately 50% membrane association and transforming activity (Kato et al., 1992). For K-Ras4B, efficient membrane binding requires both farnesylation and a polybasic domain, with carboxymethylation enhancing this interaction (Hancock et al., 1991). Farnesyltransferase inhibitors (FTIs) block H-Ras membrane localization and reverse the transformed phenotype, while a myristoylated, farnesylation-independent form is resistant (Reuveni et al., 2000). Farnesylation of K-Ras4B is also required for interaction with PDEδ, facilitating trafficking (Dharmaiah et al., 2016). A recent discovery revealed that K-Ras farnesylation at C185 drives liquid-liquid phase separation, forming condensates that promote RCE1 clustering, enhance processing, and amplify oncogenic signaling in colon cancer (Wang et al., 2026). Furthermore, PKC-mediated phosphorylation of S181 creates a farnesyl-electrostatic switch promoting dissociation from the plasma membrane and mitochondrial association, where it interacts with Bcl-XL to induce apoptosis (Bivona et al., 2006).

The functional importance extends to other Ras-related GTPases. Rheb requires farnesylation for mTORC1 activation; FTIs block this, and a farnesylation-independent Rheb mutant renders Pten-deficient tumor cells resistant to FTI therapy (Mavrakis et al., 2008). The TSC1/TSC2 complex’s GTPase activity toward Rheb depends on proper membrane localization (Tee et al., 2003). Rhes farnesylation is critical for membrane association, facilitating tunneling nanotube formation via Slc4a7 to mediate intercellular spread of mutant Huntingtin (Dagar et al., 2026). In tauopathies, Rhes farnesylation links to tau aggregation, and lonafarnib reduces tau inclusions by inhibiting Rhes farnesylation and activating autophagy (Ehrenberg et al., 2021). Rac1 undergoes farnesylation under specific conditions, such as 15(S)-HETE-induced angiogenesis, where its membrane translocation is critical for endothelial cell migration and tube formation (Nayak and Jain, 2011; Singh et al., 2011).

Subcellular targeting of Rho GTPases is determined by hypervariable regions and dynamically regulated by RhoGDI binding. RhoA, Rac1, Rac2, and Cdc42hs are regulated by RhoGDIα, whereas farnesylated RhoB and TC10 are not (Michaelson et al., 2001). RhoB localizes to the plasma membrane, Golgi, and motile peri-Golgi vesicles, while TC10 is at the plasma membrane and endosomes (Michaelson et al., 2001). Palmitoylation serves as a critical secondary targeting signal; its inhibition mislocalizes H-Ras, RhoB, and TC10 to the endoplasmic reticulum (Michaelson et al., 2001). Prenyl chain length does not affect RhoGDI binding, as substituting farnesylation for geranylgeranylation on Cdc42hs did not alter RhoGDIα interaction, whereas introducing a palmitoylation site into RhoA blocked binding entirely (Michaelson et al., 2001). Mutations rendering RhoA, Cdc42hs, or Rac1 constitutively active or dominant negative abrogate RhoGDIα binding and redirect localization to both plasma membrane and internal membranes (Michaelson et al., 2001).

### Nuclear lamins: scaffolding the nuclear envelope

Nuclear lamins are abundant farnesylated proteins. Prelamin A, lamin B1, and lamin B2 undergo farnesylation essential for nuclear envelope targeting and lamina assembly. Prelamin A processing involves farnesylation, -AAX cleavage by RCE1 or ZMPSTE24, carboxymethylation, and a second ZMPSTE24 cleavage removing the farnesylated tail to yield mature lamin A (Yao et al., 2020). Non-farnesylated prelamin A fails to assemble properly (Lutz et al., 1992). In Hutchinson-Gilford progeria syndrome (HGPS), progerin retains its farnesyl anchor due to a 50-amino acid deletion, causing nuclear blebbing, heterochromatin loss, and premature senescence (Capell et al., 2005; Yang et al., 2005; Dechat et al., 2007). FTIs improve nuclear morphology by mislocalizing progerin (Mallampalli et al., 2005; Toth et al., 2005), and lonafarnib extends survival in HGPS patients (Gordon et al., 2014; Misteli, 2021), though non-farnesylated progerin still elicits milder phenotypes (Yang et al., 2008). Farnesylated prelamin A accumulation also occurs in vascular smooth muscle cells during normal aging (Ragnauth et al., 2010).

B-type lamin farnesylation is permanent and essential. Mice expressing non-farnesylated lamin B1 die postnatally with severe neurodevelopmental defects and lamina detachment in migrating neurons (Jung et al., 2013). RCE1-mediated endoproteolysis and methylation are required for lamin B1 organization; deficiency causes nuclear lamina deformity (Maske et al., 2003). Methylation organizes lamin B1 into subdomains, and a carboxymethylation-dependent lamin receptor mediates protein–protein interactions at the nuclear periphery (Maske et al., 2003). FTIs induce donut-shaped nuclei via centrosome separation defects linked to lamin B1 farnesylation inhibition and pericentrin degradation (Verstraeten et al., 2011). BZA-5B blocks prelamin A and lamin B farnesylation with an IC50 comparable to p21ras without immediately disrupting localization (Dalton et al., 1995). Antibodies recognizing unprocessed prelamin A (Sinensky et al., 1994) and farnesylated, endoproteolyzed lamin B1 (8D1) reveal distinct pools of proteolyzed but nonmethylated and fully processed lamin B1, suggesting regulated maturation (Maske et al., 2003).

Kinetic analysis in *Xenopus* oocytes demonstrated that lamin B3 isoprenylation occurs in the cytosol before nuclear uptake; nuclear farnesylation under isoprene starvation likely represents a default pathway (Firmbach-Kraft and Stick, 1995). These findings align with dual cytosolic and nuclear protein farnesyltransferase localization (Firmbach-Kraft and Stick, 1995).

### Molecular chaperones: Hsp40/DNAJA1 and ANJ1

The Hsp40 family regulates Hsp70 ATPase activity and includes farnesylated members with specialized roles. DNAJA1, a type I Hsp40, is farnesylated at its C-terminal CaaX motif, which is essential for stabilizing activation-induced deaminase (AID). Farnesylation enables optimal DNAJA1–AID interaction, and DNAJA1 depletion reduces AID levels and isotype switching by half (Orthwein et al., 2012). In yeast, farnesylated Ydj1 (DNAJA1 ortholog) anchors to the endoplasmic reticulum membrane, confining protein deposits to the aging mother cell; membrane detachment disrupts this spatial confinement, causing premature aging in daughter cells (Saarikangas et al., 2017). The plant homolog ANJ1 is also farnesylated, enhancing microsomal membrane association and high-temperature function, as a non-farnesylatable mutant fails to complement a temperature-sensitive yeast mutant (Zhu et al., 1993). In plants, HSP40 controls meristem size, abscisic acid (ABA) signaling, and drought resistance (Barghetti et al., 2017). The Arabidopsis HSP40 proteins J2 and J3 (90% identity, C-terminal CAQQ motif) are farnesylated *in vivo*, shown by ^14^C-mevalonate labeling and gel mobility shifts dependent on farnesyl transferase (ERA1) but not geranylgeranyl transferase (GGB) (Barghetti et al., 2017). Farnesylation promotes J2/J3 membrane association, as the J3C417S mutant exhibits a higher soluble-to-membrane ratio than wild-type J3 (Barghetti et al., 2017). Transgenic expression of J3C417S in a j2/j3 double-knockout background recapitulates ABA hypersensitivity, drought resistance, late flowering, and enlarged meristem phenotypes of era1 mutants, demonstrating that defective J2/J3 farnesylation alone confers these pleiotropic effects (Barghetti et al., 2017). Loss of J2/J3 farnesylation up-regulates HSP70 and HSP90, and a synthetic genetic interaction between era1 and the HSP90 ATPase mutant hsp90.2-3 causes complete sterility and defective inflorescence development, linking protein farnesylation to the chaperone network (Barghetti et al., 2017). Furthermore, impaired J2/J3 farnesylation reduces accumulation of abiotic stress- and copper-responsive miRNAs (miR397/398/408/857) via decreased pri-miRNA transcription, likely due to compromised SPL7 function, revealing a connection between chaperone farnesylation and miRNA-mediated stress responses (Barghetti et al., 2017).

### Centromere/kinetochore proteins: orchestrating mitosis

Several kinetochore proteins require farnesylation for chromosome segregation. CENP-E farnesylation, independent of its motor activity, drives fibrous corona expansion by binding Spindly and recruiting RZZ-S complexes, promoting chromosome alignment (Wu et al., 2024). CENP-F requires farnesylation for kinetochore targeting during oocyte meiosis, essential for meiotic progression and female fertility; disruptive mutations cause oocyte maturation arrest (Zhong et al., 2026). Spindly farnesylation is essential for RZZ complex interaction and kinetochore localization. FTI treatment abrogates Spindly localization, recapitulating Spindly knockdown phenotypes and identifying Spindly as a key FTI target (Moudgil et al., 2015). The ROD subunit of RZZ serves as the farnesyl receptor for Spindly in a membrane-independent manner (Mosalaganti et al., 2017). Structural studies show RZZ is a 42-nm particle with 2:2:2 stoichiometry, where ROD’s N-terminal β-propeller binds Spindly’s C-terminal farnesyl moiety, establishing RZZ as the kinetochore receptor (Mosalaganti et al., 2017). Spindly farnesylation promotes RZZ-Spindly oligomerization into a filamentous meshwork, forming the fibrous corona’s structural basis; this occurs via hydrophobic interactions *in vitro* (Sacristan et al., 2018). The C-terminal CPQQ motif is a conserved CAAX signal; substitution with CENP-E or CENP-F motifs restores localization, whereas geranylgeranylation motifs fail, confirming farnesylation specificity and FTI sensitivity (Moudgil et al., 2015). Spindly also recruits dynein via conserved motifs (Spindly box, CC1, CC2 box) to compact kinetochores upon microtubule attachment; mutation of all three (SpindlyΔCCS) abolishes dynein recruitment, causing persistently expanded kinetochores, merotelic attachments, and segregation errors (Sacristan et al., 2018).

CENP-E depletion reduces fibrous corona size and diminishes corona-localized MAD1–MAD2 (Wu et al., 2024). The motor domain is dispensable; a C-terminal fragment (aa 2111–2701) rescues corona expansion, dependent on the CAAX motif (CKTQ). Mutation of C2698 or Q2701 abrogates this activity, and the KRAS4b CAAX box can functionally substitute, confirming farnesylation’s requirement (Wu et al., 2024). CENP-E-dependent corona expansion is critical for kinetochore-derived microtubule nucleation, as CENP-E-depleted cells show virtually no nucleation after nocodazole washout (Wu et al., 2024).

### Signaling regulators: diverse functions across compartments

Beyond the well-characterized classes above, farnesylation regulates a diverse array of signaling proteins. The peroxisomal import receptor PEX19 is farnesylated at its C-terminal CaaX motif, inducing a conformational change that creates hydrophobic pockets for peroxisomal membrane protein (PMP) recognition; mutations disrupting farnesyl contacts or PMP recognition abolish cargo binding and cannot complement PEX19 deficiency in Zellweger patient fibroblasts (Emmanouilidis et al., 2017). Farnesylation also uncouples PEX19 targeting between ER/lipid droplets and peroxisomes (Schrul and Kopito, 2016).

The deubiquitinating enzyme UCH-L1, linked to Parkinson’s disease, is farnesylated at its C-terminus, promoting membrane association. Membrane-associated, farnesylated UCH-L1 (UCH-L1M) correlates with increased intracellular α-synuclein and neurotoxicity; FTI-277 reduces UCH-L1M, α-synuclein, and increases cell viability (Liu et al., 2009). Farnesylation dictates UCH-L1 localization and regulation of mitochondrial morphology: a farnesylation-mimicking mutant enhances its effect on Mitofusin-2 (Mfn2) (Cerqueira et al., 2020). UCH-L1 knockdown reduces Mfn2 protein, leading to mitochondrial enlargement, disrupted tubular networks, reduced ER–mitochondria contacts, and impaired mitochondrial calcium uptake, while overexpression of wild-type UCH-L1 increases Mfn2; a farnesylation-site mutation (C220S) strikingly enhances this effect, indicating the soluble cytosolic form regulates Mfn2 (Cerqueira et al., 2020). UCH-L1 knockdown also elevates mitochondrial oxygen consumption rates and yields a 2.5-fold increase in Complex I activity (Cerqueira et al., 2020).

The zinc-finger antiviral protein (ZAP) long isoform requires S-farnesylation for endolysosomal targeting and antiviral activity (Charron et al., 2013). The SNARE Ykt6 is dually lipidated: farnesylation is a prerequisite for subsequent palmitoylation, stably anchoring it to membranes for intra-Golgi transport and viability (Fukasawa et al., 2004), and acts as a conformational switch stabilizing an autoinhibited closed state (Wen et al., 2010). Complexins III and IV are C-terminally farnesylated, regulating synaptic targeting and transmitter release, and can functionally replace non-farnesylated complexins I and II (Reim et al., 2005). Nitric oxide-mediated S-nitrosylation of complexin modulates its farnesylation-dependent clamping ability, suppressing evoked and spontaneous release by reducing vesicle pools; S-nitrosylation alters membrane association and enhances SNARE interaction, antagonizing farnesylation as a nitrergic molecular switch (Robinson et al., 2018).

PRL tyrosine phosphatases (PRL-1, -2, -3) require farnesylation to promote cell invasion and motility via Rho GTPase activation (Fiordalisi et al., 2006). PARIS (ZNF746) farnesylation prevents its accumulation and neurodegeneration in Parkinson’s models; enhancing farnesylation via farnesol protects against aging-related muscle weakness (Jo et al., 2021; Bae et al., 2023).

Rnd3 farnesylation at C241 cooperates with phosphorylation to regulate 14-3-3ζ binding. Phosphorylated S240 is the essential gatekeeper, while farnesylated C241 and additional phosphorylation at S210/S218 enhance interaction through distinct mechanisms: the farnesyl moiety binds a hydrophobic groove on one 14-3-3ζ monomer, inducing pS240 binding via an induced-fit mechanism with positive cooperativity, while remote phosphosites mediate multivalent binding across the dimer; the farnesyl group also acts as a kinetic stabilizer decelerating complex dissociation (Hu et al., 2021).

The striatal-enriched GTPase Rhes (Rasd2) requires farnesylation for membrane anchoring and tunneling nanotube (TNT) formation; a C263S mutant fails to promote TNT biogenesis (Dagar et al., 2026). Farnesylation-dependent binding to the sodium bicarbonate cotransporter Slc4a7 modulates intracellular pH to facilitate TNT formation and mutant Huntingtin (mHTT) transfer; Slc4a7 depletion or knock-out reduces TNT formation and mHTT transmission *in vivo*, highlighting a therapeutic target in Huntington’s disease (Dagar et al., 2026).

In host-pathogen interactions, the *Legionella pneumophila* effector AnkB is farnesylated by host machinery, anchoring it to the Legionella-containing vacuole for docking polyubiquitinated proteins and generating amino acids for proliferation (Price et al., 2010; 2011). Effector DenR hijacks host farnesylated NRas to the vacuole surface, rewiring signaling to downregulate MAPK (Lehman et al., 2024).

## Farnesylation in ras signaling and oncogenesis

### The central role of farnesylation in Ras membrane targeting and trafficking

**FIGURE 3:**
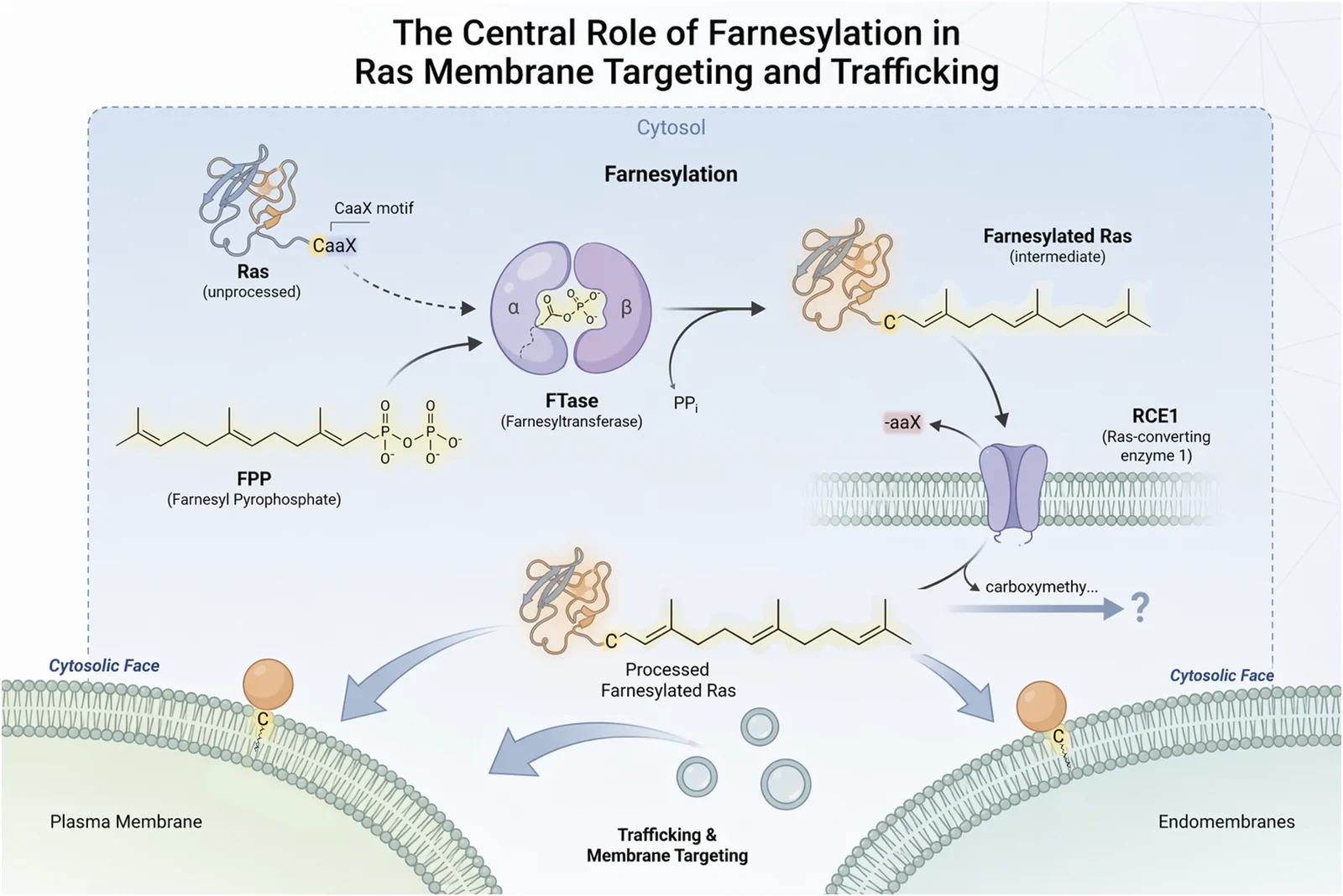
Post-translational processing and membrane targeting of Ras proteins driven by farnesylation. Newly synthesized, unprocessed Ras resides in the cytosol and features a C-terminal CaaX motif. The initial and rate-limiting step of lipid modification is catalyzed by the heterodimeric Farnesyltransferase (FTase), which covalently attaches a 15-carbon farnesyl isoprenoid from Farnesyl Pyrophosphate (FPP) to the cysteine residue of the CaaX box, releasing inorganic pyrophosphate (PPi). The farnesylated Ras intermediate is then targeted to intracellular membranes (typically the endoplasmic reticulum), where Ras-converting enzyme 1 (RCE1) endoproteolytically cleaves the terminal three amino acids (-aaX). Following this cleavage, the newly exposed isoprenylated cysteine typically undergoes carboxymethylation to form the fully processed farnesylated Ras. Finally, the mature Ras protein is trafficked via vesicular transport to its ultimate cellular destinations, anchoring firmly into the lipid bilayers of the plasma membrane or various endomembranes to exert its biological signaling functions.

Farnesylation is the obligate first step in post-translational processing of all Ras isoforms. (Casey et al., 1989; Reiss et al., 1990). This modification is catalyzed by farnesyltransferase (FTase), a heterodimeric enzyme transferring farnesyl from farnesyl pyrophosphate (FPP) (Goodman et al., 1990; He et al., 1991). Farnesylation is essential for subsequent processing—proteolytic removal of the -aaX tripeptide by Ras-converting enzyme 1 (RCE1) and carboxymethylation by isoprenylcysteine carboxyl methyltransferase (ICMT)—and for proper membrane association and biological activity (Hancock et al., 1991; Kato et al., 1992) (Figure 3; Table 3).

**TABLE 3:** Key findings on farnesylation in Ras membrane targeting and trafficking.

Name	Function	Citation
Farnesylation	Obligate first step in Ras processing; covalent attachment of 15-carbon farnesyl lipid to CaaX cysteine	Casey et al. (1989), Reiss et al. (1990)
Farnesyltransferase (FTase)	Heterodimeric enzyme catalyzing farnesyl transfer from FPP to Ras	Goodman et al. (1990), He et al. (1991)
RCE1	Proteolytic removal of -aaX tripeptide after farnesylation	Hancock et al. (1991), Kato et al. (1992)
ICMT	Carboxymethylation of farnesylated cysteine for proper membrane association	Hancock et al. (1991), Kato et al. (1992)
RAM1/RAM2	Yeast FTase β and α subunits; mutants lack FTase activity and fail Ras processing/localization	Goodman et al. (1990), He et al. (1991)
Farnesylated but non-proteolyzed/non-methylated Ki-Ras mutant	Retained ∼50% membrane binding and full transforming potential	Kato et al. (1992)
Farnesylated but unmethylated K-Ras(B)	Binds membranes inefficiently, showing complete processing needed for plasma membrane targeting	Hancock et al. (1991)
GGPPS	Catalyzes GGPP synthesis at mevalonate pathway branch point; altered activity shifts farnesylation/geranylgeranylation balance	Wang et al. (2013)
GGPPS-deficient Sertoli cells	Exhibit constitutively activated MAPK signaling via altered Ras prenylation	Wang et al. (2013)

Early biochemical and genetic studies established farnesylation’s critical importance. Purified FTase transfers farnesyl from FPP to Ras CaaX sequences, abolished by cysteine mutations in the motif (Manne et al., 1990; Reiss et al., 1990). Yeast mutants defective in RAM1 or RAM2 (encoding FTase β and α subunits) lack FTase activity and fail to process and localize Ras (Goodman et al., 1990; He et al., 1991). A farnesylated but non-proteolyzed, non-methylated Ki-Ras mutant retained ∼50% membrane binding and full transforming potential (Kato et al., 1992), yet complete processing is required for efficient plasma membrane targeting, as farnesylated but unmethylated K-Ras(B) binds membranes inefficiently (Hancock et al., 1991).

Beyond the canonical pathway, interplay between farnesylation and geranylgeranylation regulates Ras membrane targeting. Geranylgeranyl pyrophosphate synthase (GGPPS) catalyzes synthesis of geranylgeranyl pyrophosphate (GGPP), the 20-carbon isoprenoid donor, at a mevalonate pathway branch point also producing FPP (Wang et al., 2013). Altered GGPPS activity shifts the farnesylation/geranylgeranylation balance, impacting Ras processing. GGPPS-deficient Sertoli cells exhibit constitutively activated MAPK signaling via altered Ras prenylation, as Ras can be modified by both GGPP and FPP depending on CaaX motif properties (Wang et al., 2013). Thus, Ras farnesylation fidelity depends not solely on FTase activity but also on relative isoprenoid substrate availability, linking cellular metabolic states to Ras membrane targeting and downstream signaling regulation.

### Isoform-specific trafficking: H-Ras, K-Ras, and lipid raft dynamics

**FIGURE 4:**
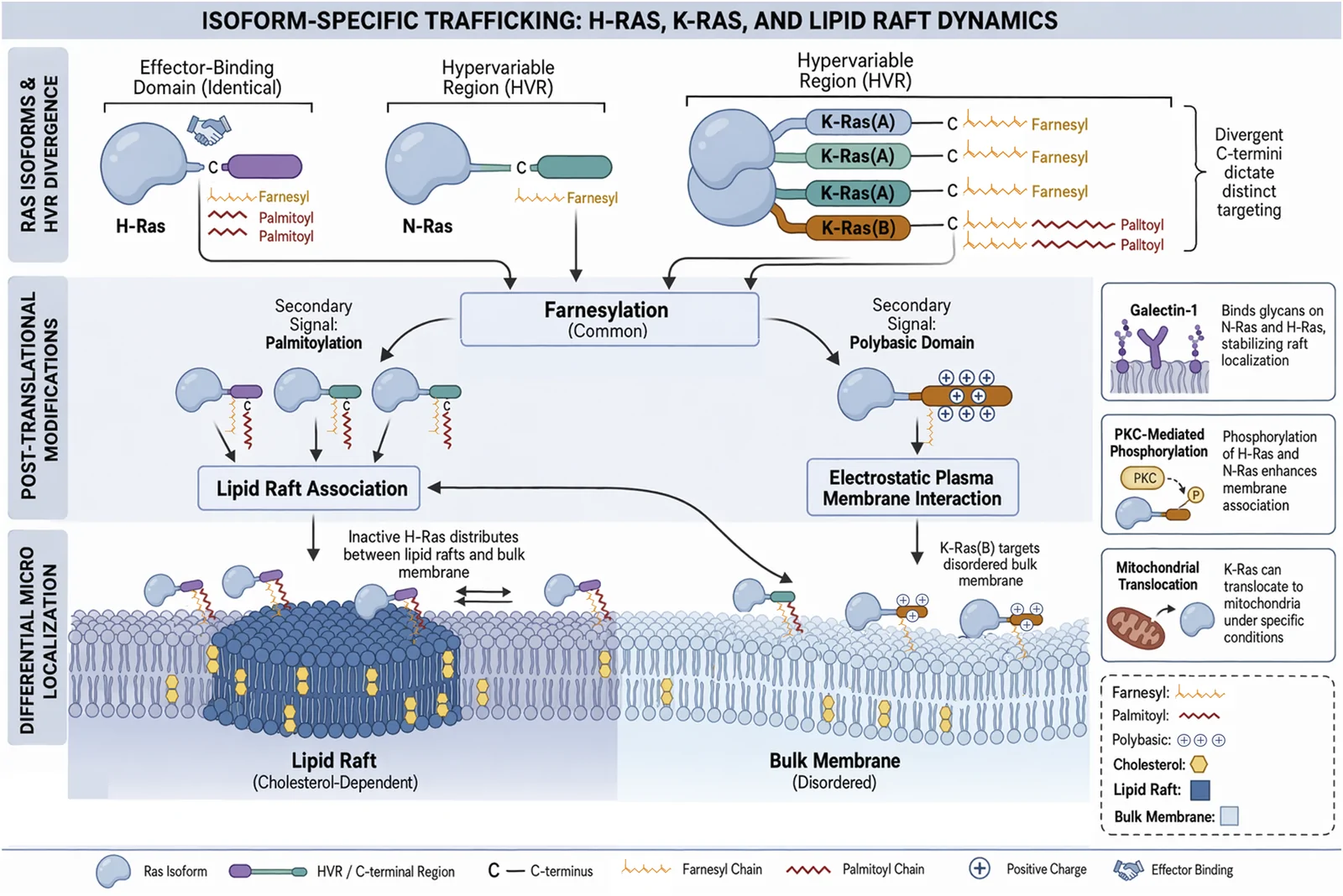
Structural divergence in the hypervariable region (HVR) dictates isoform-specific post-translational modifications and differential spatial organization of Ras proteins at the plasma membrane (Top) Isoform Divergence: While all major Ras isoforms (H-Ras, N-Ras, and K-Ras splice variants A/B) share an identical and highly conserved N-terminal effector-binding domain, they possess highly divergent C-terminal hypervariable regions (HVRs). This structural divergence acts as the primary determinant for their distinct intracellular targeting pathways (Middle) Divergent Secondary Signals: Following a universal and mandatory primary farnesylation step (yielding a generic membrane affinity), the distinct HVRs provide unique secondary targeting signals. Isoforms such as H-Ras, N-Ras, and K-Ras(A) undergo secondary, reversible palmitoylation, which acts as a signal for lipid raft association. In stark contrast, K-Ras(B) relies on a polybasic domain—a cluster of positively charged amino acids—serving as an alternative secondary signal that promotes strong electrostatic interactions with the negatively charged inner leaflet of the plasma membrane (Bottom) Differential Microdomain Localization: These distinct lipid and electrostatic modifications segregate Ras isoforms into specific lateral microdomains within the plasma membrane. Palmitoylated isoforms (e.g., inactive H-Ras) dynamically partition between highly ordered, cholesterol-dependent lipid rafts and the bulk membrane. Conversely, K-Ras(B), driven by its farnesyl anchor and polybasic domain, is generally excluded from lipid rafts and exclusively targets the disordered, non-raft bulk membrane environment.

The three major Ras isoforms share identical effector-binding domains but diverge in their C-terminal hypervariable regions (HVRs), dictating distinct membrane targeting. All require farnesylation, but secondary signals differ: H-Ras, N-Ras, and K-Ras(A) undergo palmitoylation, while K-Ras(B) utilizes a polybasic lysine domain for electrostatic plasma membrane interaction (Kaplan and Sass, 1991) (Figure 4; Table 4).

**TABLE 4:** Isoform-specific membrane targeting and microdomain localization of Ras isoforms.

Name	Function	Citation
H-Ras, N-Ras, K-Ras(A)	Undergo palmitoylation for membrane targeting	Kaplan and Sass (1991)
K-Ras(B)	Utilizes a polybasic lysine domain for electrostatic plasma membrane interaction	Kaplan and Sass (1991)
Inactive H-Ras	Distributes between cholesterol-dependent lipid rafts and cholesterol-independent microdomains	Prior et al. (2001), 2003
GTP-bound H-Ras	Exits lipid rafts; GTP-dependent segregation critical for Raf activation	Prior et al. (2001), 2003
Lipid raft microdomains (GFP-tH)	Mean radius of 22 ± 4 nm; occupy ∼35% of the plasma membrane	Prior et al. (2003)
H-RasG12 V	Constitutively active; shows no raft colocalization	Prior et al. (2003)
K-Ras	Localizes outside lipid rafts irrespective of nucleotide state; occupies distinct cholesterol-independent microdomains (mean radius 16 ± 3 nm, ∼20% of membrane)	Prior et al. (2003)
Galectin-1	Stabilizes activated H-Ras in non-raft microdomains; depletion reduces H-RasG12 V clustering	Prior et al. (2003)
PKC-mediated phosphorylation of K-Ras serine 181	Farnesyl-electrostatic switch promoting dissociation from plasma membrane and translocation to mitochondria	Bivona et al. (2006)
Mitochondrial K-Ras	Interacts with Bcl-XL to promote apoptosis	Bivona et al. (2006)
Farnesyl-to-geranylgeranyl replacement on K-Ras	Strikingly reduces K-Ras clustering; farnesylation specifically supports nanocluster formation	Prior et al. (2003)

These mechanisms yield differential microdomain localization. Inactive H-Ras distributes between cholesterol-dependent lipid rafts and cholesterol-independent microdomains, whereas GTP-bound H-Ras exits rafts (Prior et al., 2001; 2003). Lipid raft microdomains (GFP-tH) have a mean radius of 22 ± 4 nm and occupy ∼35% of the plasma membrane (Prior et al., 2003). GDP-bound H-Ras colocalizes with GFP-tH, but this decreases upon serum stimulation; constitutively active H-RasG12 V shows no raft colocalization (Prior et al., 2003). This GTP-dependent segregation is critical for Raf activation (Prior et al., 2001). Conversely, K-Ras localizes outside lipid rafts irrespective of nucleotide state, occupying distinct cholesterol-independent microdomains (mean radius 16 ± 3 nm, ∼20% of membrane); cholesterol depletion slightly increases K-Ras clustering (Prior et al., 2003). Galectin-1 stabilizes activated H-Ras in non-raft microdomains, as its depletion reduces H-RasG12V clustering (Prior et al., 2003).

K-Ras localization is further regulated by PKC-mediated phosphorylation of serine 181, a farnesyl-electrostatic switch promoting dissociation from the plasma membrane and translocation to mitochondria, where it interacts with Bcl-XL to promote apoptosis (Bivona et al., 2006). The prenyl group critically influences organization: replacing farnesyl with geranylgeranyl strikingly reduces K-Ras clustering, indicating farnesylation specifically supports nanocluster formation (Prior et al., 2003).

### Effector engagement and downstream signaling

Farnesylation directly influences Ras-effector interactions. The farnesylated yeast Ras2 exhibits >100-fold higher affinity for adenylyl cyclase compared to unprocessed forms (Kuroda et al., 1993) (Table 5). Similarly, smg p21 GDS is active exclusively on post-translationally processed c-Ki-Ras and RhoA, demonstrating that C-terminal processing is a prerequisite for functional regulatory protein interactions (Mizuno et al., 1991).

**TABLE 5:** Ras effector engagement and downstream signaling: Key findings.

Name	Function	Citation
Farnesylated yeast Ras2	>100-fold higher affinity for adenylyl cyclase vs. unprocessed forms	Kuroda et al. (1993)
smg p21 GDS	Active only on processed c-Ki-Ras and RhoA; C-terminal processing required for regulatory interactions	Mizuno et al. (1991)
Membrane-targeted Sos derivatives	Farnesylation/myristoylation signals sufficient for oncogenic transformation	Aronheim et al. (1994)
Membrane-targeted RafCAAX	Constitutively active independently of Ras	Stokoe et al. (1994)
L-744,832 (FTI)	Selectively inhibits H-Ras-transformed cell proliferation; loss of farnesylated H-Ras and EGFR ligand downregulation	Sizemore et al. (1999)
HR12 (FTI)	Reverses Ras-induced transformed phenotype; restores focal adhesions, stress fibers, adherens junctions via MEK/ERK	Reuveni et al. (2000)
HR12 effect on Erk1and2	Dose- and time-dependent decline in activation (t_1_/_2_–6 h); correlates with nonfarnesylated oncogenic Ras accumulation	Reuveni et al. (2000)
Myristylated Ras + HR12	HR12 fails to restore nontransformed phenotype, confirming specificity for farnesylation inhibition	Reuveni et al. (2000)
PD98059 (MEK inhibitor)	Induces cytoskeletal recovery indistinguishable from HR12	Reuveni et al. (2000)
Constitutively active MEK	Prevents HR12-induced cytoskeletal effects	Reuveni et al. (2000)
LY294002 (PI3K inhibitor)	No cytoskeletal restoration; establishes MEK/ERK as dominant pathway	Reuveni et al. (2000)
Tipifarnib (FTI)	Induces apoptosis in lymphoid cells via Bim upregulation through c-Raf–MEK1/2–ERK1/2 inhibition	Ding et al. (2011)

The primary Ras effectors—Raf kinases and PI3K p110—are recruited to the plasma membrane upon activation. Membrane-targeted Sos derivatives containing farnesylation or myristoylation signals are sufficient for oncogenic transformation (Aronheim et al., 1994), while a membrane-targeted RafCAAX construct is constitutively active independently of Ras (Stokoe et al., 1994). Thus, farnesylated, membrane-bound Ras serves as a platform for effector recruitment and activation.

The Ras-Raf-MEK-ERK and Ras-PI3K-Akt cascades drive proliferation, survival, and transformation. FTIs block these outputs by preventing farnesylation and membrane association. L-744,832 selectively inhibits H-Ras-transformed cell proliferation, correlating with loss of farnesylated H-Ras and downregulation of EGFR ligands (Sizemore et al., 1999). HR12 reverses the Ras-induced transformed phenotype by restoring focal adhesions, stress fibers, and adherens junctions via the MEK/ERK pathway (Reuveni et al., 2000). HR12 treatment induces a dose- and time-dependent decline in Erk1&2 activation (t_1_/_2_ ∼6 h), correlating with nonfarnesylated oncogenic Ras accumulation (Reuveni et al., 2000). Critically, HR12 fails to restore the nontransformed phenotype in cells expressing myristylated Ras, confirming specificity for farnesylation inhibition (Reuveni et al., 2000). The MEK inhibitor PD98059 induces cytoskeletal recovery indistinguishable from HR12, while constitutively active MEK prevents HR12-induced effects; the PI3K inhibitor LY294002 produces no such restoration, establishing MEK/ERK as the dominant pathway (Reuveni et al., 2000). Additionally, tipifarnib induces apoptosis in lymphoid cells via Bim upregulation through c-Raf–MEK1/2–ERK1/2 inhibition (Ding et al., 2011).

### Farnesylation-dependent Ras activation in oncogenic transformation and tumor maintenance

Oncogenic Ras dependence on farnesylation drove FTI development. Peptidomimetic FTIs selectively reverse Ras-transformed morphology without affecting Raf- or Mos-transformed cells (James et al., 1993; Kohl et al., 1993). L-731,734 inhibited Ras processing and colony formation in v-ras-transformed cells but not v-raf or v-mos counterparts (Kohl et al., 1993), while B956 inhibited anchorage-independent growth across human tumor lines with various ras oncogenes (Nagasu et al., 1995) (Table 6).

**TABLE 6:** Key studies on farnesylation-dependent ras activation and therapeutic interventions.

Name	Function	Citation
Peptidomimetic FTIs (L-731,734, B956)	Selectively reverse Ras-transformed morphology; inhibit Ras processing and colony formation in v-ras cells but not v-raf/v-mos; inhibit anchorage-independent growth in human tumor lines with ras oncogenes	James et al. (1993), Kohl et al. (1993), Nagasu et al. (1995)
FTI-276	Selectively blocks growth of human lung carcinoma with K-Ras mutation and p53 deletion in nude mice; suppresses Ras processing	Sun et al. (1995)
SCH66336	Inhibits Bcr/Abl-positive leukemia growth in P190 transgenic mice where Ras is activated downstream of Bcr/Abl	Reichert et al. (2001)
R115777 (tipifarnib)	Antitumor activity in MCF-7 xenografts; correlates with unfarnesylated prelamin A accumulation; induces complete remission in 14% of older adults with poor-risk AML; stabilizes disease in 64% of multiple myeloma patients	Kelland et al. (2001), Alsina et al. (2004), Lancet et al. (2007)
FTI-277	Suppresses Sca-1-positive cancer stem cell maintenance in syngeneic breast cancer model with elevated activated Ras and phosphorylated MEK	Kim et al. (2010)
FTase genetic ablation	FTase essential for embryonic development but dispensable for adult homeostasis; limited effect on Ras-dependent tumor progression; combined FTase/GGTase-I inactivation markedly reduces K-RAS-induced lung tumor formation	Mijimolle et al. (2005), Liu et al. (2010)
BMS-214662	Induces apoptosis in B-CLL cells including nucleoside analog-resistant cases via mitochondrial membrane potential loss independent of Akt	Marzo et al. (2004)
Salirasib (FTS)	Disrupts Ras–membrane interactions; lacks significant efficacy in Panc-1 xenografts	Xiao et al. (2014)
Rasfonin	2-pyrone derivative; selectively kills K-ras-mutated Panc-1 cells (IC50 = 5.5 μM) over wild-type BxPC-3 cells (IC50 = 10 μM); reduces Ras–GTP and c-Raf/MEK/ERK phosphorylation via reduced Sos1; reduces Panc-1 xenograft tumor growth by 67%	Xiao et al. (2014)

**FIGURE 5:**
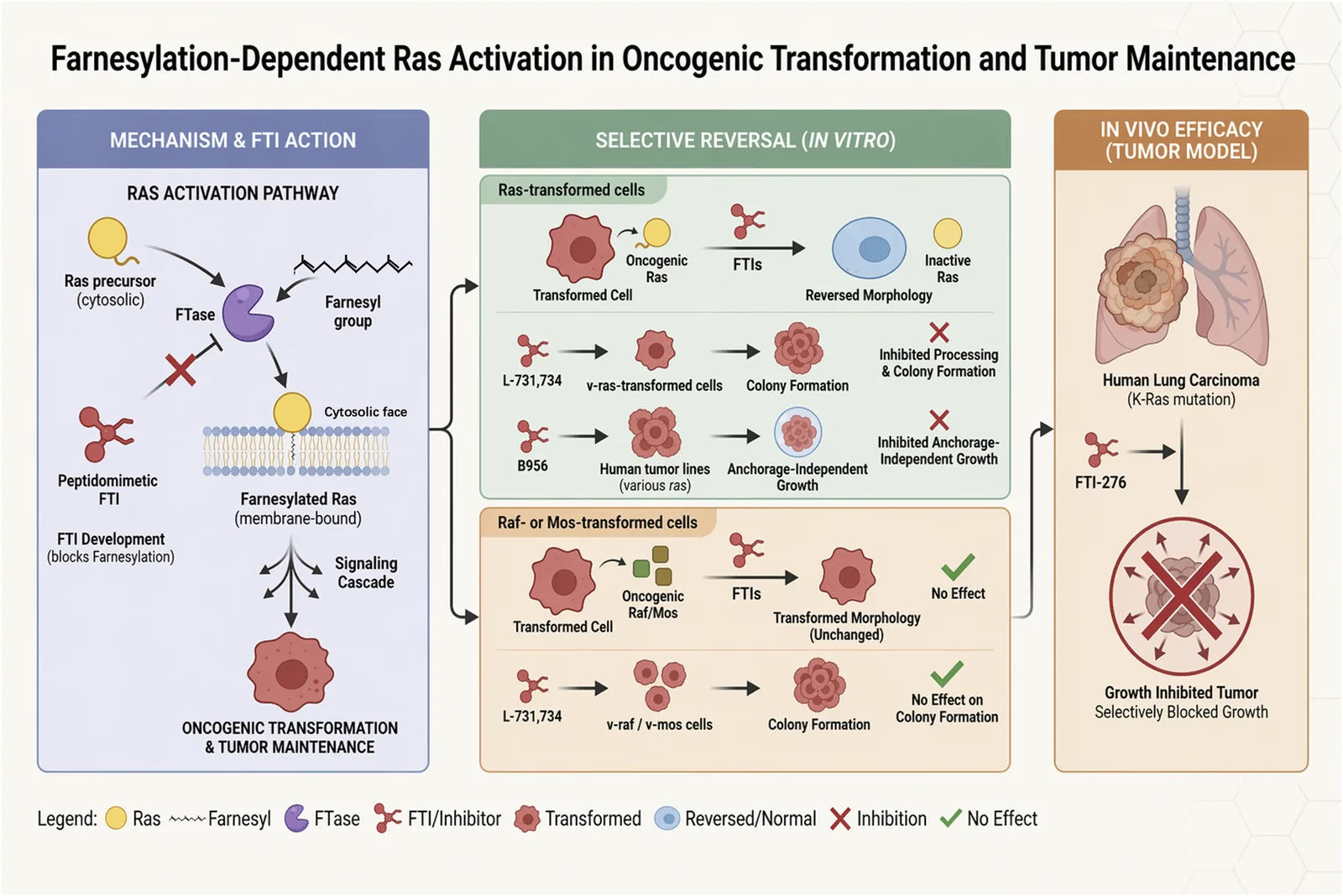
Farnesyltransferase inhibitors (FTIs) as a targeted therapeutic strategy: From biochemical mechanism to *in vitro* selectivity and *in vivo* tumor suppression (Left) Mechanism of FTI Action: The oncogenic activity of mutated Ras variants is strictly dependent on membrane localization driven by farnesylation. Peptidomimetic FTIs are rationally designed to competitively inhibit the enzyme FTase, thereby blocking the transfer of the farnesyl group to the cytosolic Ras precursor. This pharmacological blockade prevents Ras membrane association, aborts downstream oncogenic signaling cascades, and ultimately halts transformation and tumor maintenance (Middle) Selective Reversal *In Vitro*: The efficacy of FTIs demonstrates high pathway selectivity in cellular models. In Ras-transformed cells (e.g., driven by v-ras or human tumor lines with various ras mutations), treatment with FTIs (such as L-731,734 or B956) successfully reverts the malignant morphological phenotype to a normalized state and robustly inhibits anchorage-independent colony formation. Crucially, these FTIs exhibit no significant effect on cells transformed by downstream or alternative oncogenes (such as Raf or Mos), underscoring the specific requirement of FTase activity for Ras-mediated pathogenesis (Right) *In Vivo* Efficacy in Tumor Models: The biochemical and cellular selectivity translates into profound anti-tumor efficacy *in vivo*. In preclinical models, such as human lung carcinoma harboring K-Ras mutations, systemic administration of FTIs (e.g., FTI-276) selectively and potently inhibits tumor growth, validating FTase as a viable therapeutic target for Ras-driven malignancies.

*In vivo*, FTI-276 selectively blocked growth of a human lung carcinoma with K-Ras mutation and p53 deletion in nude mice, correlating with suppressed Ras processing (Sun et al., 1995). SCH66336 inhibited Bcr/Abl-positive leukemia growth in P190 transgenic mice, where Ras is activated downstream of Bcr/Abl (Reichert et al., 2001). R115777 (tipifarnib) showed antitumor activity in MCF-7 xenografts, correlating with unfarnesylated prelamin A accumulation (Kelland et al., 2001). FTI-277 suppressed Sca-1-positive cancer stem cell maintenance in a syngeneic breast cancer model, where these cells exhibited elevated activated Ras and phosphorylated MEK (Kim et al., 2010) (Figure 5).

Genetic studies revealed FTase is essential for embryonic development but dispensable for adult homeostasis (Mijimolle et al., 2005). FTase ablation in Ras-dependent tumor models had limited effect on progression, suggesting farnesylation contributes to but is not absolutely required for tumor maintenance (Mijimolle et al., 2005). In K-RAS-induced lung cancer, FTase inactivation reduced tumor growth and improved survival; combined FTase and GGTase-I inactivation markedly reduced tumor formation (Liu et al., 2010).

In hematologic malignancies, tipifarnib induced complete remission in 14% of older adults with poor-risk AML, with HDJ-2 farnesylation inhibition observed (Lancet et al., 2007). In multiple myeloma, tipifarnib stabilized disease in 64% of patients and inhibited FTase activity and survival pathways (Alsina et al., 2004). BMS-214662 induced apoptosis in B-CLL cells from all patients, including nucleoside analog-resistant cases, via mitochondrial membrane potential loss independent of Akt (Marzo et al., 2004).

Alternative strategies include Salirasib (FTS), which disrupts Ras–membrane interactions. Rasfonin, a 2-pyrone derivative, selectively killed K-ras-mutated Panc-1 cells (IC50 = 5.5 μM) over wild-type K-ras BxPC-3 cells (IC50 = 10 μM) and suppressed contact-independent growth, migration, and invasion dose-dependently (Xiao et al., 2014). Rasfonin reduced Ras–GTP levels and downstream c-Raf/MEK/ERK phosphorylation without affecting farnesylation, partially via reduced Sos1 expression (Xiao et al., 2014). In Panc-1 xenografts, rasfonin (30 mg/kg) reduced tumor growth by 67% (T/C = 33%, P = 0.019) and halved final tumor weight, whereas FTS lacked significant efficacy (P = 0.337) (Xiao et al., 2014).

### Alternative prenylation of K-Ras and N-Ras: resistance mechanisms to FTase inhibition

A major challenge in targeting Ras through FTase inhibition is alternative prenylation. While H-Ras is exclusively farnesylated, K-Ras and N-Ras can undergo geranylgeranylation by GGTase-I when FTase is inhibited, retaining membrane association and biological activity (James et al., 1996; Lobell et al., 2001). The FTI BZA-5B failed to block prenylation of a chimeric H/K-RasBV12 protein or reverse transformed morphology, and L-739,749 failed to inhibit K-RasB prenylation in Rat1 cells (James et al., 1996).

The C-terminal CaaX X residue determines specificity: methionine or serine directs farnesylation, leucine or isoleucine directs geranylgeranylation (Finegold et al., 1991; Kinsella et al., 1991). K-Ras and N-Ras terminate with methionine (CVIM and CVVM), making them FTase substrates, but under FTI treatment they become GGTase-I substrates. K-Ras remains prenylated in FTI-treated cells via GGTase-I modification, and Ki-ras-transformed cells are more resistant to FTIs than Ha-ras-transformed cells (Lobell et al., 2001). Combining an FTI with a GGTI or using dual prenylation inhibitors (DPIs) overcomes this resistance, as shown in human PSN-1 pancreatic tumor cells harboring oncogenic Ki-ras (Lobell et al., 2001).

Dual FTase and GGTase-I inhibitors have been developed to address this resistance. FGTI-2734, a RAS C-terminal mimetic dual inhibitor, blocked KRAS membrane localization in pancreatic, lung, and colon cancer cells and induced apoptosis, whereas selective FTIs or GGTIs alone were ineffective (Kazi et al., 2019). FGTI-2734, but not FTI-2148 or GGTI-2418, blocked KRAS membrane association by immunofluorescence, cellular fractionation, and gel shift assays, and inhibited growth of patient-derived pancreatic tumors in mice (Kazi et al., 2019). It was highly effective against primary and metastatic mutant KRAS tumor cells from eight pancreatic cancer patients in three-dimensional co-cultures with pancreatic stellate cells, suppressed AKT, mTOR, and cMYC pathways, upregulated p53, and induced apoptosis in patient-derived xenografts *in vivo* (Kazi et al., 2019). The dual inhibitor L-778,123 was evaluated clinically with radiotherapy for locally advanced pancreatic cancer, showing feasibility but dose-limiting gastrointestinal toxicities (Martin et al., 2004).

Alternative prenylation has also been observed with RCE1 deficiency. Inactivation of Rce1 in mouse fibroblasts mislocalizes RAS and inhibits transformation, yet paradoxically accelerates K-RAS-induced myeloproliferative disease *in vivo*, suggesting complex context-dependent effects (Wahlstrom et al., 2007).

### Therapeutic implications and clinical development of farnesyltransferase inhibitors

FTIs including tipifarnib, lonafarnib, and BMS-214662 have undergone extensive clinical evaluation (Table 7). Phase I studies established safety profiles: tipifarnib showed dose-limiting myelosuppression with FTase activity and HDJ-2 farnesylation inhibition as biomarkers (Zimmerman et al., 2004; Widemann et al., 2006); lonafarnib caused gastrointestinal toxicity and fatigue, with prelamin A farnesylation inhibition confirming target engagement (Adjei et al., 2000); BMS-214662 exhibited gastrointestinal and hematologic toxicities (Ryan et al., 2004).

**TABLE 7:** Clinical evaluation and therapeutic applications of farnesyltransferase inhibitors (FTIs).

Agent/Regimen	Indication & phase	Key outcome	Citation
Phase I — safety, pharmacokinetics, and PD biomarkers			
Tipifarnib (R115777)	Phase I, advanced solid tumors	Dose-limiting myelosuppression; FTase activity and HDJ-2 farnesylation inhibition as PD biomarkers	Zimmerman et al. (2004), Widemann et al. (2006)
Lonafarnib (SCH66336)	Phase I, advanced malignancies	GI toxicity and fatigue; prelamin A farnesylation inhibition confirms target engagement	Adjei et al. (2000)
BMS-214662	Phase I, advanced solid tumors	GI and hematologic toxicities; broad-spectrum cytotoxicity	Ryan et al. (2004)
L-778,123 (dual FTase/GGTase-I)	Phase I, advanced malignancies	Continuous-infusion infusion; protein farnesylation inhibition confirmed in PBMCs	Britten et al. (2001)
Lonafarnib	Phase I, pediatric CNS tumors	Dose-limiting pneumonitis and myelosuppression; HDJ-2 farnesylation inhibition measured	Kieran et al. (2007)
BMS-214662 + paclitaxel/carboplatin	Phase I, advanced solid tumors	FTase inhibition demonstrated via unfarnesylated HDJ-2 accumulation	Dy et al. (2005)
Tipifarnib + capecitabine	Phase I, advanced solid tumors	Combination feasible; capecitabine does not alter tipifarnib pharmacology	Gore et al. (2006)
Phase II — single agent, hematologic malignancies			
Tipifarnib	Phase II, refractory acute leukemias	Inhibits FTase and farnesylation of lamin A and HDJ-2; clinical responses in refractory AML	Karp et al. (2001)
Tipifarnib	Phase II, poor-risk older AML	Complete remission 14%; overall response 23%	Lancet et al. (2007)
Tipifarnib	Phase II, relapsed/refractory multiple myeloma	Stable disease in 64%; FTase inhibited in bone marrow	Alsina et al. (2004)
Lonafarnib	Phase II, MDS/CMML	24% response rate with decreased HDJ-2 farnesylation	Feldman et al. (2008)
Phase II — single agent, solid tumors			
Tipifarnib	Phase II, advanced NSCLC	No objective responses; median survival 7.7 months; ∼16% disease stabilization >6 months	Adjei et al. (2003b)
Tipifarnib	Phase II, metastatic pancreatic adenocarcinoma	No objective responses despite FTase inhibition	Cohen et al. (2003)
Tipifarnib	Phase II, advanced breast cancer	Partial responses in 10%–14%	Johnston et al. (2003)
Tipifarnib	Phase II, sensitive-relapse SCLC	Limited activity; median PFS 1.4 months	Heymach et al. (2004)
Tipifarnib	Phase II, NF1 plexiform neurofibromas	No prolongation of time to progression	Widemann et al. (2014)
Phase II — genotype-matched indication			
HRAS-mutant selectivity (rationale)	Mechanistic rationale	HRAS, unlike K-/N-Ras, lacks an alternative-prenylation backup and is uniquely dependent on FTase; preclinical selective mislocalisation and viability loss in HRAS-mutant models	Gilardi et al. (2020)
Tipifarnib (AIM-HN trial)	Phase II, HRAS-mutant HNSCC	Objective responses in ∼50% of patients with high HRAS mutant-allele frequency; first validated biomarker-selected indication for an FTI	Ho et al. (2021)
Phase II — combinations, solid tumors			
Tipifarnib + gemcitabine/cisplatin	Phase I/II, advanced solid tumors	Combination feasible; prelamin A accumulation confirms FTase inhibition	Adjei et al. (2003a)
Tipifarnib + gemcitabine	Phase II, pancreatic cancer	Clinical activity with HDJ-2 farnesylation inhibition	Patnaik et al. (2003)
Approved indication			
Lonafarnib (Zokinvy)	HGPS (FDA approval)	Improves cardiovascular, bone, and survival outcomes; significant survival extension vs. matched historical controls	Gordon et al. (2014), 2016; Misteli (2021)

Phase II results were mixed. Tipifarnib showed no objective responses in non-small-cell lung cancer (median survival 7.7 months) (Adjei et al., 2003b) or pancreatic adenocarcinoma (Cohen et al., 2003), 10%–14% partial responses in breast cancer (Johnston et al., 2003), and limited activity in small-cell lung cancer (median PFS 1.4 months) (Heymach et al., 2004). Single-agent efficacy is limited, likely due to alternative K-Ras prenylation and target complexity. AIMP2-DX2, a splicing variant regulating KRAS stability by blocking Smurf2-mediated degradation upstream of farnesylation, highlights additional regulatory mechanisms (Kim et al., 2022).

Combination strategies enhanced efficacy: tipifarnib with gemcitabine/cisplatin showed feasibility (Adjei et al., 2003a) and with gemcitabine demonstrated clinical activity with HDJ-2 farnesylation inhibition (Patnaik et al., 2003). Lonafarnib with paclitaxel synergistically enhanced tubulin acetylation and mitotic arrest (Marcus et al., 2005). FTIs synergize with microtubule-stabilizing agents by enhancing mitotic sensitivity (Moasser et al., 1998) and FTI-277 radiosensitized H-ras-transformed cells (Bernhard et al., 1996). Pharmacological targeting of the AIMP2-DX2–KRAS interface reduces KRAS levels and suppresses KRAS-dependent growth (Kim et al., 2022).

In Hutchinson-Gilford progeria syndrome (HGPS), FTIs improve nuclear abnormalities and disease phenotypes (Capell et al., 2005; Mallampalli et al., 2005; Yang et al., 2005). Lonafarnib is approved for HGPS, improving cardiovascular, bone, and survival outcomes (Gordon et al., 2014; 2016; Misteli, 2021). Combined statin/aminobisphosphonate therapy more effectively inhibits both farnesylation and geranylgeranylation (Varela et al., 2008).

In tauopathies, lonafarnib reduces tau inclusions and atrophy by inhibiting Rhes farnesylation, causing Rhes to sequester Beclin-1 and upregulate autophagy (Ehrenberg et al., 2021). Postmortem studies across sporadic tauopathies reveal tau-specific Rhes distribution changes, and single-nucleus RNA sequencing confirms Rhes expression in cortical neurons vulnerable in tauopathies, supporting clinical application of lonafarnib (Ehrenberg et al., 2021).

### Farnesylation beyond Ras: broader implications in cancer and cellular signaling

While Ras proteins are the most studied farnesylated substrates, FTase modifies a diverse array of proteins, and the antitumor effects of FTIs likely involve targets beyond Ras. The farnesylated proteins include nuclear lamins (prelamin A and lamin B), the chaperone protein HDJ-2, the peroxisomal import receptor PEX19, the SNARE protein Ykt6, the mitotic checkpoint protein Spindly, the centromere protein CENP-E, and the Rheb GTPase, among others (Dalton et al., 1995; Mavrakis et al., 2008; Wen et al., 2010; Moudgil et al., 2015; Emmanouilidis et al., 2017; Wu et al., 2024) (Table 8). The development of chemical biology tools, such as the tagging-via-substrate (TAS) technology using azido-farnesyl analogs, has enabled global profiling of farnesylated proteins, revealing the breadth of the farnesylation proteome (Kho et al., 2004). More recently, quantitative chemical proteomics using isoprenoid analogues YnF and YnGG have identified 80 prenylated proteins in a single human cell line, enabling system-wide analysis of prenylation dynamics and alternative prenylation in response to prenyltransferase inhibitors (Storck et al., 2019).

**TABLE 8:** Farnesylated proteins and their functions beyond ras.

Name	Function	Citation
Nuclear lamins (prelamin A, lamin B)	Farnesylated structural proteins of the nuclear lamina	Dalton et al. (1995)
HDJ-2	Farnesylated chaperone protein	Dalton et al. (1995)
PEX19	Farnesylated peroxisomal import receptor	Wen et al. (2010)
Ykt6	Farnesylated SNARE protein	Wen et al. (2010)
Spindly	Farnesylated mitotic checkpoint protein; essential for kinetochore localization and RZZ complex interaction; key FTI target in mitosis	Moudgil et al. (2015)
CENP-E	Farnesylated centromere protein; drives fibrous corona expansion at kinetochores independent of motor activity	Wu et al. (2024)
Rheb	Farnesylated GTPase and mTORC1 activator; required for oncogenic activity; FTI target in lymphomagenesis	Mavrakis et al. (2008)
PARIS (ZNF746)	Farnesylated transcriptional repressor of PGC-1α; farnesylation prevents PGC-1α repression; decreased farnesylation in Parkinson’s disease substantia nigra; neuroprotective role	Jo et al. (2021)
Ras and RhoB	Farnesylated GTPases; inhibition by tipifarnib contributes to protective effects in pulmonary hypertension	Duluc et al. (2017)

Most recently, this chemical-proteomic strategy has been extended to a quantitative, system-wide map covering more than twelve thousand proteins across lung-cancer cell lines, simultaneously defining the farnesylated proteome at unprecedented depth and charting tipifarnib-driven relocalisation on a proteome scale (Pan et al., 2025). This analysis nominated INPP5A farnesylation as a selectable vulnerability in NRAS- and GNAQ/GNA11-mutant melanoma and uncovered synergistic killing when tipifarnib was combined with ferroptosis induction, opening a chemically tractable axis that links farnesylation-dependent membrane remodelling to lipid-peroxidation vulnerability (Pan et al., 2025). Work of this scale transforms the open problem of “which farnesylated substrate matters in which tumour context” into an empirically answerable question, and we expect it to motivate the next wave of biomarker-selected FTI indications.

Notably, the parkin-interacting substrate PARIS (ZNF746), a transcriptional repressor of PGC-1α implicated in Parkinson’s disease pathogenesis, was recently identified as a novel farnesylated protein (Jo et al., 2021). PARIS contains an atypical C-terminal CaaX motif (631CGLS634) that is recognized by FTase, and its farnesylation at cysteine 631 was confirmed by tandem mass spectrometry, metabolic labeling with azido-farnesyl alcohol, and *in vitro* farnesylation assays (Jo et al., 2021). Farnesol, a natural isoprenoid, promotes PARIS farnesylation in a dose-dependent manner, which prevents PARIS-mediated transcriptional repression of PGC-1α by decreasing PARIS occupancy on the PPARGC1A promoter (Jo et al., 2021). This modification is physiologically relevant, as PARIS farnesylation is decreased in the substantia nigra of patients with Parkinson’s disease, and conditional knockout of the FTase β subunit in mouse substantia nigra blocks PARIS farnesylation, leading to PGC-1α reduction (Jo et al., 2021). These findings expand the functional repertoire of protein farnesylation to include neuroprotective roles in neurodegenerative disease.

The farnesylation of Rheb, a proximal activator of mTORC1, is required for its oncogenic activity, and FTIs can block Rheb-driven lymphomagenesis (Mavrakis et al., 2008). Farnesylation of the mitotic checkpoint protein Spindly is essential for its kinetochore localization and interaction with the RZZ complex, and FTI treatment phenocopies Spindly knockdown, indicating that Spindly is a key FTI target in mitosis (Moudgil et al., 2015). The farnesylation of CENP-E drives the expansion of the fibrous corona at kinetochores, a function independent of its motor activity (Wu et al., 2024). These findings highlight that the anticancer effects of FTIs may be mediated through multiple farnesylated substrates involved in cell cycle regulation, mitosis, and signal transduction.

Beyond oncology, protein farnesylation has emerged as a critical mediator in pulmonary vascular disease. Increased levels of protein farnesylation have been detected in lung tissues from rodent models of pulmonary hypertension and from patients with pulmonary arterial hypertension (PAH) (Duluc et al., 2017). The orally bioavailable FTI tipifarnib prevented the development of hypoxia-induced pulmonary hypertension in mice, reducing right ventricular systolic pressure and right ventricular hypertrophy, and attenuating pulmonary vascular muscularization (Duluc et al., 2017). Mechanistically, tipifarnib reduced hypoxia-induced proliferation of human pulmonary artery endothelial cells and smooth muscle cells, increased endothelium-dependent vasodilation, and reduced vasoconstriction of intrapulmonary arteries without affecting cell viability (Duluc et al., 2017). These protective effects were associated with inhibition of Ras and RhoB farnesylation, actin depolymerization, and increased endothelial nitric oxide synthase (eNOS) expression *in vitro* and *in vivo* (Duluc et al., 2017). Notably, RhoB is unique among Rho proteins in that it can be either farnesylated (F-RhoB) or geranylgeranylated (GG-RhoB), and its biological activity is determined by its prenylation status (Duluc et al., 2017). Farnesylated-only RhoB promoted proliferative responses in cultured pulmonary vascular cells, mimicking the effects of hypoxia, whereas geranylgeranylated-only RhoB and tipifarnib exerted inhibitory effects (Duluc et al., 2017). Label-free proteomics linked F-RhoB with cell survival, cell cycle activation, and mitochondrial biogenesis, and hypoxia increased the levels of F-RhoB-regulated proteins in the lung, an effect reduced by tipifarnib (Duluc et al., 2017). Importantly, unlike statins, which inhibit both farnesylation and geranylgeranylation by depleting mevalonate-derived isoprenoids, tipifarnib did not increase the expression levels of Rho proteins, suggesting a more selective mode of action (Duluc et al., 2017). These findings provide a rationale for selectively targeting protein farnesylation in pulmonary hypertension and expand the therapeutic potential of FTIs to cardiovascular diseases.

The differential impact of inhibiting farnesylation versus geranylgeranylation has been further elucidated in the context of innate immune cell function. In a study comparing the effects of statins on freshly isolated human monocytes versus monocyte-derived macrophages, inhibition of geranylgeranylation, but not farnesylation, was found to mediate the retainment of cytokine production capacity in macrophages differentiated in the presence of statin (Fu et al., 2019). Specifically, while statin treatment did not alter cytokine production (TNF-α, IL-6, IL-1β) in LPS-stimulated freshly isolated monocytes, macrophages differentiated overnight in the absence of statin lost the capacity to produce these cytokines, whereas macrophages differentiated in the presence of statin retained robust cytokine production—an effect termed the “retainment effect” (Fu et al., 2019). Reconstitution experiments demonstrated that mevalonic acid, farnesyl pyrophosphate (FPP), and geranylgeranyl pyrophosphate (GGPP) all blocked the retainment effect, whereas squalene, a cholesterol synthesis intermediate, did not, confirming that the isoprenylation pathway, rather than cholesterol synthesis, was responsible (Fu et al., 2019). Critically, pharmacological inhibition with the geranylgeranyl transferase inhibitor GGTI-298 mimicked the statin effect, whereas the farnesyl transferase inhibitor FTI-277 did not, demonstrating that geranylgeranylation, not farnesylation, is the key prenylation event regulating this functional switch during monocyte-to-macrophage differentiation (Fu et al., 2019). Furthermore, the retainment effect was shown to be mediated by enhanced Rac1 activation (Rac1-GTP levels), as inhibition of Rac1 by the Rac1/TIAM1 inhibitor NSC23766 or by Rac1-siRNA blocked the statin-induced retainment of cytokine production (Fu et al., 2019). These findings reveal a cell-type-specific role for geranylgeranylation-dependent Rac1 signaling in regulating macrophage functional maturation and highlight the distinct biological consequences of selectively targeting farnesylation versus geranylgeranylation in immune cells (Fu et al., 2019).

In the context of cancer stem cells, Ras farnesylation contributes to the maintenance and expansion of Sca-1-positive putative cancer stem cells in a mouse model of breast cancer, and the FTI FTI-277 suppresses this population, suggesting that selective inhibition of Ras activation may be useful for stem cell-specific cancer therapy (Kim et al., 2010). The mevalonate pathway and protein farnesylation also regulate the replication of oncolytic viruses, and inhibition of this pathway with FTIs enhances the anticancer potency of M1 virus (Liang et al., 2018).

In summary, farnesylation is a critical post-translational modification that governs the membrane association, subcellular trafficking, and effector engagement of Ras proteins, thereby controlling their oncogenic signaling output. While the development of FTIs as Ras-targeted therapeutics has been complicated by alternative prenylation of K-Ras and N-Ras, these agents have shown clinical activity in hematologic malignancies and have emerged as a life-extending therapy for HGPS. The broader farnesylation proteome and the development of dual prenylation inhibitors continue to offer opportunities for therapeutic intervention in Ras-driven cancers and other diseases. The recent discovery that PARIS farnesylation regulates PGC-1α activity and protects against dopaminergic neurodegeneration further illustrates that farnesylation impacts diverse pathophysiological processes beyond oncology, suggesting that pharmacological modulation of farnesylation may have therapeutic potential in neurodegenerative disorders (Jo et al., 2021). Moreover, the demonstration that tipifarnib prevents hypoxia-induced pulmonary hypertension by targeting F-RhoB-mediated proliferative signaling and vascular remodeling extends the therapeutic relevance of FTIs to pulmonary vascular disease (Duluc et al., 2017). The differential roles of farnesylation and geranylgeranylation in immune cell differentiation further underscore the importance of prenylation specificity, as geranylgeranylation-dependent Rac1 activation, rather than farnesylation, controls the functional maturation of macrophages, suggesting that selective prenyltransferase inhibitors may have distinct immunomodulatory applications (Fu et al., 2019).

**FIGURE 6:**
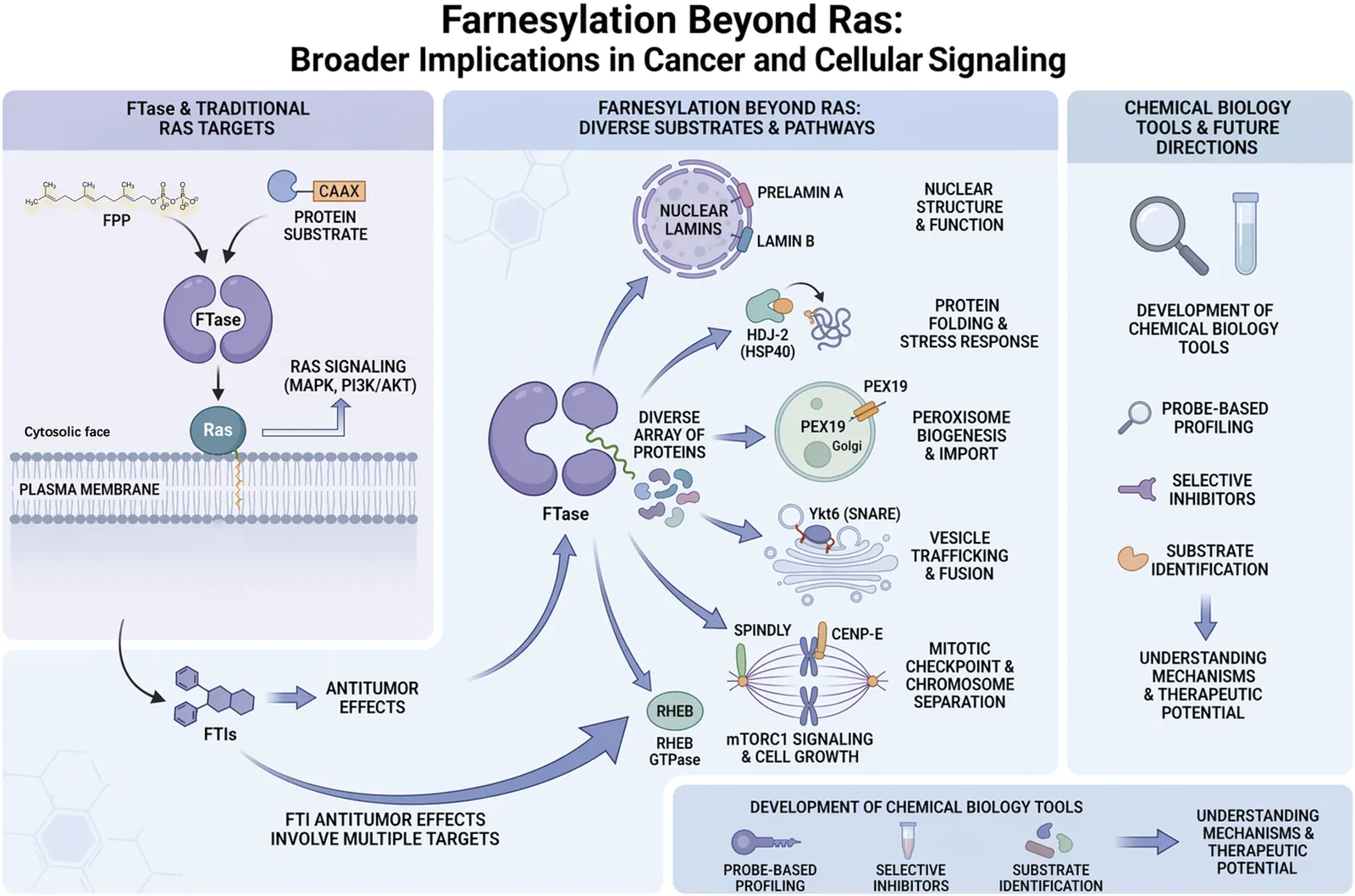
The expanding landscape of protein farnesylation: from canonical Ras targeting to diverse cellular networks and future therapeutic strategies (Left) The canonical FTase–Ras paradigm. Farnesyltransferase (FTase) catalyzes the covalent addition of a 15-carbon isoprenyl group from farnesyl pyrophosphate (FPP) to the cysteine residue of proteins containing a C-terminal CAAX motif. Historically, Ras GTPases have been the most extensively studied substrates, where farnesylation is obligate for membrane localization and subsequent activation of oncogenic signaling cascades (e.g., MAPK, PI3K/AKT). Farnesyltransferase inhibitors (FTIs) were originally designed to selectively block this process (Center) Farnesylation beyond Ras. Accumulating evidence reveals a much broader “farnesylome.” FTase acts upon a diverse array of crucial cellular proteins, thereby regulating multiple physiological and pathological pathways. These include maintaining nuclear architecture (Prelamin A, Lamin B), facilitating protein folding and stress responses (HDJ-2/HSP40 chaperone), mediating peroxisome biogenesis (PEX19), orchestrating SNARE-dependent vesicle trafficking (Ykt6), ensuring proper mitotic spindle assembly and chromosome segregation (Spindly, CENP-E), and regulating cell growth via mTORC1 signaling (Rheb). Consequently, the clinical antitumor efficacy of FTIs is now understood to be pleiotropic, deriving from the simultaneous inhibition of this multiplexed substrate network rather than solely Ras blockade (Right & Bottom) Chemical biology tools and future directions. To fully delineate the farnesylation network and exploit it therapeutically, the field is increasingly relying on advanced chemical biology approaches. The development of probe-based metabolic profiling (e.g., clickable lipid analogs), generation of highly selective inhibitors, and robust substrate identification platforms (chemoproteomics) are critical. These tools will deepen our mechanistic understanding of prenylation biology and uncover novel, non-Ras therapeutic vulnerabilities for cancer treatment.

On our read, the most tractable near-term therapeutic directions to emerge from this broader proteome are the dual FTase/GGTase-I inhibitors in KRAS-driven tumours, genotype-matched FTI use in HRAS-mutant cancers, INPP5A-directed exploration of NRAS/GNAQ/GNA11-mutant melanoma, and PARIS/farnesol strategies in Parkinson’s disease; the principal uncertainty remains proteome-wide target deconvolution in patient-derived contexts, without which the field will continue to struggle to nominate indications beyond HRAS (Figure 6).

### Nuclear lamins, progeria, and the farnesylation–aging nexus

Lamin A, a key structural component of the nuclear lamina, is synthesized as a precursor protein, prelamin A, which undergoes a complex series of post-translational modifications at its C-terminal CAAX motif. This processing is essential for its proper localization and function. The initial and obligate step is the addition of a 15-carbon farnesyl isoprenoid lipid to the cysteine residue of the CAAX motif by the enzyme farnesyltransferase (FTase) (Manne et al., 1990; Reiss et al., 1990). This farnesylation event is not unique to prelamin A; it is a critical modification for a wide array of proteins, including the Ras family of small GTPases, where it is essential for membrane association and biological activity (Casey et al., 1989; Kato et al., 1992). Following farnesylation, the terminal three amino acids (the -AAX) are cleaved by the endoprotease RAS-converting enzyme 1 (RCE1) or the zinc metalloproteinase ZMPSTE24 (Ashby et al., 1992; Maske et al., 2003). The newly exposed C-terminal farnesylcysteine is then methylated by the enzyme isoprenylcysteine carboxyl methyltransferase (ICMT) (Hancock et al., 1991). In the final, unique step of lamin A maturation, ZMPSTE24 performs a second endoproteolytic cleavage 15 amino acids upstream of the farnesylated cysteine, removing the modified tail and releasing mature, non-farnesylated lamin A (Lutz et al., 1992). This final cleavage step is crucial, as it distinguishes lamin A from other permanently farnesylated CAAX proteins like lamin B1, where farnesylation is important for its retention of nuclear chromatin during neuronal migration (Jung et al., 2013) (Table 9).

**TABLE 9:** Key post-translational modifications and processing enzymes in lamin a maturation and progeria.

Name	Function	Citation
Farnesyltransferase (FTase)	Enzyme that adds a 15-carbon farnesyl isoprenoid lipid to the cysteine residue of the CAAX motif of prelamin A and other proteins (e.g., Ras family)	Manne et al. (1990), Reiss et al. (1990)
RAS-converting enzyme 1 (RCE1)/ZMPSTE24	Endoproteases that cleave the terminal three amino acids (-AAX) after farnesylation	Ashby et al. (1992), Maske et al. (2003)
Isoprenylcysteine carboxyl methyltransferase (ICMT)	Enzyme that methylates the newly exposed C-terminal farnesylcysteine	Hancock et al. (1991)
ZMPSTE24 (second cleavage)	Performs a second endoproteolytic cleavage 15 amino acids upstream of the farnesylated cysteine, releasing mature, non-farnesylated lamin A	Lutz et al. (1992)
Progerin	Truncated prelamin A variant in HGPS caused by *LMNA* c.1824C>T mutation; retains CAAX motif, undergoes farnesylation and carboxymethylation, but lacks the second ZMPSTE24 cleavage site, resulting in permanent farnesylation and nuclear membrane anchorage	Capell et al. (2005), Cao et al. (2007), Dechat et al. (2007)
Permanently farnesylated lamin A mutants (progerin, LA L647 R)	Induce heterochromatin loss, replication-dependent DNA damage, proliferation defects, and premature senescence; toxicity driven by permanent farnesyl moiety, not the 50-amino acid deletion	Foo et al. (2024)
Non-farnesylated SSIM mutants	CAAX cysteine mutated to serine; do not induce the cellular defects seen with permanently farnesylated mutants, demonstrating that farnesylation is the sole driver of toxicity	Foo et al. (2024)

The pathological consequences of defects in this processing pathway are profoundly illustrated by Hutchinson–Gilford progeria syndrome (HGPS), a rare, fatal premature aging disease. HGPS is most commonly caused by a *de novo* point mutation in the LMNA gene (c.1824C>T) that activates a cryptic splice donor site, leading to the production of a truncated prelamin A variant termed progerin (Capell et al., 2005). Progerin retains the CAAX motif and undergoes farnesylation and carboxymethylation, but the internal 50-amino acid deletion removes the second ZMPSTE24 cleavage site. Consequently, progerin remains permanently farnesylated and anchored to the inner nuclear membrane (Cao et al., 2007; Dechat et al., 2007). This permanent anchorage is the primary driver of its toxicity, as it is the farnesylation, and not the deletion itself, that is solely responsible for progerin-induced cellular defects and its rapid accumulation (Foo et al., 2024). A systematic dissection of the relative contributions of the Δ50 A A versus permanent farnesylation, using a panel of lamin A mutants expressed in normal human dermal fibroblasts, demonstrated that permanently farnesylated lamin A mutants (progerin and LA L647R, which cannot be cleaved by ZMPSTE24) induced heterochromatin loss (H3K9me3 and H3K27me3), replication-dependent DNA damage, proliferation defects, and premature senescence, whereas their non-farnesylated SSIM counterparts (in which the CAAX cysteine is mutated to serine) did not, establishing that the permanent farnesyl moiety, and not the 50-amino acid deletion, is the sole driver of these cellular phenotypes (Foo et al., 2024). The accumulation of progerin disrupts the integrity of the nuclear lamina, leading to hallmark nuclear blebbing, heterochromatin loss, impaired nucleocytoplasmic transport, and DNA damage accumulation, ultimately driving cells into premature senescence (Yang et al., 2005; Balmus et al., 2018; Chang et al., 2019). Clinically, cardiovascular disease in HGPS is characterized by early and pervasive vascular stiffening and later-stage arterial occlusive disease, which culminate in premature death from heart attack or, less often, stroke (Gordon et al., 2014). The median life expectancy at birth for HGPS patients is 14.6 years, with cardiovascular abnormalities—including medial smooth-muscle cell loss, adventitial fibrosis, and depletion of smooth-muscle actin—representing the major pathologies contributing to morbidity and lethality (Gordon et al., 2014; Balmus et al., 2018).

The central role of farnesylation in progerin toxicity has made it a prime therapeutic target. Farnesyltransferase inhibitors (FTIs), originally developed to block the oncogenic activity of Ras proteins (James et al., 1993; Kohl et al., 1993), have been shown to mislocalize progerin away from the nuclear envelope, dramatically improving nuclear shape abnormalities in HGPS fibroblasts (Capell et al., 2005; Mallampalli et al., 2005). This finding was extended to other progeroid syndromes, such as restrictive dermopathy caused by ZMPSTE24 deficiency, where FTIs similarly ameliorate nuclear blebbing by preventing the accumulation of farnesylated prelamin A (Toth et al., 2005). The clinical translation of this strategy has been a landmark in progeria therapeutics. The FTI lonafarnib has been shown to improve cardiovascular and bone disease, and critically, to significantly extend survival in children with HGPS (Gordon et al., 2014; 2016; Misteli, 2021). A pivotal study comparing survival in treated versus age- and gender-matched untreated HGPS cohorts demonstrated a hazard ratio of 0.13 (95% CI 0.04–0.37; P < 0.001), with 21 of 43 deaths in the untreated group versus five of 43 deaths among treated subjects over a median follow-up of 5.3 years from treatment initiation, translating to an increase in mean survival of 1.6 years (Gordon et al., 2014). This study also established a robust natural history of untreated HGPS, with a mean survival of 14.6 years (Gordon et al., 2014). A plasma progerin assay has been developed to monitor therapeutic response, demonstrating that lonafarnib treatment reduces circulating progerin levels (Gordon et al., 2023). This ultrasensitive single-molecule counting immunoassay, with a linear dynamic detection range of 59 to 30,000 pg/mL and no cross-reactivity to lamin A, revealed that mean plasma progerin in drug-naive HGPS patients (n = 74) was 33,261 ± 12,346 pg/mL, representing a 95-fold increase over non-HGPS controls (351 ± 251 pg/mL; P < 0.0001), with no differences by sex (Gordon et al., 2023). Lonafarnib treatment resulted in an average per-visit progerin decrease from baseline of 35%–62% (all P < 0.005), with levels falling within 4 months of therapy and remaining lower for up to 10 years; critically, the magnitude of progerin decrease positively associated with patient survival (P < 0.0001), such that a 15,000 pg/mL decrease yields a 63.9% decreased risk of death, and for any given decrease in progerin, life expectancy incrementally increased with longer treatment duration (Gordon et al., 2023). However, the clinical benefit of FTIs is incomplete, partly because nonfarnesylated progerin can also elicit disease phenotypes, albeit milder ones (Yang et al., 2008), and because alternative prenylation by geranylgeranyltransferase can occur when FTase is inhibited (Varela et al., 2008). This has led to combination therapies using statins and aminobisphosphonates to inhibit both farnesylation and geranylgeranylation, which have shown improved phenotypes in mouse models (Varela et al., 2008). A subsequent clinical trial evaluated the combination of lonafarnib with pravastatin and zoledronic acid in 37 children with HGPS, demonstrating that 71.0% of participants achieved the pre-specified primary outcome of improved per-patient rate of weight gain or carotid artery echodensity (P < 0.0001), with no participants withdrawing due to side effects (Gordon et al., 2016). This triple therapy provided additional bone mineral density benefit, with significant increases in areal (P = 0.001) and volumetric (P < 0.001–0.006) bone mineral density and 1.5–1.8-fold increases in radial bone structure (P < 0.001); however, comparisons with lonafarnib monotherapy revealed likely no added cardiovascular benefit, as evidenced by no significant changes in median carotid artery wall echodensity or carotid-femoral pulse wave velocity, and increases in the percentages of participants with carotid artery plaques (5%–50%; P = 0.001) and extraskeletal calcifications (34.4%–65.6%; P = 0.006) (Gordon et al., 2016). Consistent with this, the addition of pravastatin and zoledronate did not augment the progerin-lowering effect of lonafarnib (Gordon et al., 2023).

Beyond targeting progerin processing directly, other approaches, such as inhibiting the N-acetyltransferase 10 (NAT10) with the small molecule Remodelin, have been shown to enhance healthspan in a progeria mouse model by ameliorating nuclear shape and chromatin organization defects (Balmus et al., 2018). In the LmnaG609G/G609G HGPS mouse model, oral administration of Remodelin led to a 25% increase in Kaplan–Meier area under the curve based on 20% body weight loss, and significantly reduced the loss of subcutaneous adipose tissue, adventitial fibrosis of the aorta, vascular smooth muscle cell loss, and smooth muscle actin depletion in the aorta and coronary arteries (Balmus et al., 2018). Furthermore, genetic reduction of Nat10 gene dosage in LmnaG609G/G609G mice similarly extended the timeframe of body-weight loss, validating NAT10 as the relevant pharmacological target mediating these *in vivo* effects (Balmus et al., 2018). Furthermore, targeting RCE1 has emerged as a potential strategy, as its knockout can delay senescence and improve progeria-like phenotypes in ZMPSTE24 deficiency (Yao et al., 2020). In ZMPSTE24-deficient fibroblasts from a patient with non-classical HGPS, RCE1 knockout delayed senescence and increased proliferation, an effect not observed in classical LMNA-mutant HGPS cells, indicating that the therapeutic benefit of RCE1 targeting is specific to contexts where prelamin A processing is blocked at the ZMPSTE24 step (Yao et al., 2020). *In vivo*, knockout of Rce1 in Zmpste24-deficient mice at postnatal week 4–5 increased body weight and doubled the median survival time, demonstrating a robust survival benefit (Yao et al., 2020). Mechanistically, RCE1 deficiency did not correct nuclear shape abnormalities but reduced the interaction between prelamin A and AKT, thereby activating AKT-mTOR signaling, which was required for the increased proliferation; furthermore, prelamin A levels increased in Rce1-deficient cells due to a slower turnover rate, yet its localization at the nuclear rim was unaffected (Yao et al., 2020). These findings strengthen the concept that phenotypic improvement can occur independently of nuclear shape correction and highlight RCE1 as a promising therapeutic target for progeroid disorders associated with ZMPSTE24 deficiency (Yao et al., 2020).

The continued search for novel inhibitors of protein farnesylation has been facilitated by high-throughput screening strategies leveraging the unique properties of pluripotent stem cells. A large-scale drug screen of 21,608 small molecules was performed on mesenchymal stem cells derived from HGPS induced pluripotent stem cells (HGPS MSCs), using the nuclear accumulation of prelamin A as a readout for farnesylation inhibition, with a robust Z′ factor superior to 0.8 (Blondel et al., 2016). This screen identified 11 validated hits with greater than 50% efficacy and no cellular toxicity, including six aminopyrimidines (mono-APs, di-APs, and tetra-APs), two quinoline carboxamides, one statin (simvastatin), one indole surrogate, and one spirochromane, with EC50 values ranging from 1 to 34 μM (Blondel et al., 2016). Among these, the mono-aminopyrimidines (Mono-AP1, Mono-AP2, and Mono-AP3) emerged as the most effective compounds for rescuing nuclear shape abnormalities and premature osteogenic differentiation in HGPS MSCs, while exhibiting less cytostatic activity than the FTI tipifarnib in long-term live imaging cultures (Blondel et al., 2016). Structure–activity relationship analysis of 47 Mono-AP1 analogs revealed that the piperazine nucleus, the basicity of its N4 nitrogen, and the nitrile residue are critical for biological activity, and identified two second-generation compounds, Mono-AP21 and Mono-AP28, with improved EC50 values of 1.5 and 2 μM, respectively (Blondel et al., 2016). Mechanistically, the mono-APs function as dual inhibitors targeting two key enzymes of the farnesylation pathway: farnesyl pyrophosphate synthase (FPPS) and farnesyltransferase (FT), but not HMG-CoA reductase (Blondel et al., 2016). Molecular docking and surface plasmon resonance studies confirmed direct binding of Mono-AP1 and Mono-AP2 to FPPS, with the phenyl domain mediating cation–pi interactions with the magnesium cation in the FPPS active site, while Mono-AP1 additionally showed hydrogen bonding with FT residues Y861B and S599B (Blondel et al., 2016). Enzymatic assays demonstrated that mono-APs directly inhibited both FPPS (40%–50% decrease) and FT (20%–30% decrease), with IC50 values ranging from 0.35 to 9.32 μM (Blondel et al., 2016). This dual inhibition of sequential steps in the protein prenylation pathway represents a novel pharmacological strategy that may offer therapeutic advantages over FTase-specific inhibitors alone, and these aminopyrimidines may also have broader applicability to cancers and other diseases involving farnesylated proteins (Blondel et al., 2016).

Mechanistic studies have provided critical insight into how FTIs exert their therapeutic effects at the cellular level. Using a doxycycline-inducible system to control the expression of progerin and various lamin A mutants, it was demonstrated that FTI treatment (FTI-277) does not accelerate the clearance of already farnesylated progerin; rather, FTI directly prevents progerin farnesylation, thereby significantly reducing its accumulation rate, an effect that is dependent on the farnesylatable cysteine residue, as progerin SSIM levels remained unaffected by FTI treatment (Foo et al., 2024). This finding establishes that the primary mechanism of FTI action in HGPS is the prevention of newly synthesized progerin from acquiring its toxic farnesyl modification, rather than promoting the removal of existing farnesylated progerin. Importantly, permanently farnesylated lamin A forms (progerin and LA L647R) exhibit both faster accumulation and slower clearance compared to their non-farnesylated counterparts, indicating that the farnesyl moiety directly regulates protein turnover dynamics (Foo et al., 2024). These observations suggest that early FTI intervention, before substantial progerin accumulation occurs, would be most effective, consistent with the clinical experience that lonafarnib treatment provides greater benefit when initiated earlier in the disease course.

The pathological accumulation of farnesylated prelamin A is not confined to rare progeroid syndromes; it is also a feature of physiological vascular aging. In aged vascular smooth muscle cells (VSMCs), prelamin A accumulates, leading to nuclear morphology defects, cellular senescence, and calcification, a process that can be reversed by FTI or statin treatment (Ragnauth et al., 2010). This accumulation is observed in medial VSMCs from aged individuals and in atherosclerotic lesions, establishing prelamin A as a novel biomarker of human vascular aging. Notably, plasma progerin is detectable in non-HGPS individuals at low levels (mean 351 pg/mL) and does not correlate with age (r = 0.3, P = 0.81), nor is it elevated in patients with congestive heart failure or kidney disease, suggesting its potential utility as a biomarker for studying generalized aging and atherosclerosis (Gordon et al., 2023). The connection between lamin A processing and vascular health extends to other laminopathies. For instance, mutations in LMNA that cause familial partial lipodystrophy (FPLD2) also lead to prelamin A accumulation in endothelial cells, causing endothelial dysfunction and contributing to the severe early atherosclerosis observed in these patients (Bidault et al., 2013). Similarly, HIV protease inhibitors, a cornerstone of antiretroviral therapy, have been shown to inhibit ZMPSTE24, leading to the accumulation of farnesylated prelamin A, oxidative stress, and premature cellular senescence in fibroblasts and mesenchymal stem cells, which may underlie the lipodystrophy and metabolic side effects associated with these drugs (Caron et al., 2007; Coffinier et al., 2007; Hernandez-Vallejo et al., 2013). The farnesylation-dependent anchoring of prelamin A to the nuclear envelope can also serve as a nucleation platform for signaling complexes, as demonstrated by the recruitment of RIPK1 to the nucleus in ZMPSTE24-deficient cells, which triggers necroptosis and inflammation (Yang et al., 2024).

Beyond its role in pathological contexts, farnesylated prelamin A serves critical physiological functions in tissue development and homeostasis. In differentiating human skeletal muscle, farnesylated prelamin A accumulates specifically in committed myoblasts and myotubes, where it is absent from cycling myoblasts, indicating a differentiation-dependent regulation of prelamin A processing (Mattioli et al., 2011). This accumulation of farnesylated prelamin A triggers the upregulation and recruitment of SUN1 to the nuclear envelope, while also favoring the enrichment of SUN2 at the nuclear poles (Mattioli et al., 2011). Mechanistically, farnesylated prelamin A both transcriptionally upregulates SUN1 expression (approximately threefold) and stabilizes SUN1 protein at the nuclear envelope by protecting it from proteasomal and lysosomal degradation, as demonstrated by the fact that overexpression of a farnesylated prelamin A mutant unable to bind SUN1 (LA L647R/R527P) fails to increase SUN1 staining despite inducing SUN1 mRNA (Mattioli et al., 2011). Importantly, impairment of prelamin A farnesylation by mevinolin, an HMG-CoA reductase inhibitor, abolishes SUN1 recruitment to the nuclear envelope and disrupts the polarized localization of SUN2 at the nuclear poles, leading to severe defects in myonuclear positioning, with nuclei clustering at the extremities of multinucleated myotubes (Mattioli et al., 2011). This farnesylated prelamin A–SUN1/SUN2 axis is essential for proper nuclear anchorage and spacing during myogenesis, and its disruption may contribute to the pathogenesis of muscle laminopathies. Indeed, in myoblasts from patients with autosomal dominant Emery–Dreifuss muscular dystrophy (EDMD2), reduced levels of farnesylated prelamin A and SUN1 are observed, concomitant with nuclear clustering and misshaping in differentiated myotubes, recapitulating the phenotype seen upon pharmacological inhibition of farnesylation in normal muscle cells (Mattioli et al., 2011). Furthermore, SUN1 and prelamin A levels undergo parallel age-related modulation in human skeletal muscle, with faint or absent labeling in myofibers from young individuals (4 months) and progressively increased staining from adulthood (age 20) through advanced age (age 82), suggesting that the prelamin A–SUN1 complex also participates in muscle homeostasis and the adaptation of nucleo-cytoskeletal interactions during aging (Mattioli et al., 2011).

The broader implications of protein farnesylation in aging and disease are underscored by its role in diverse cellular processes. The mevalonate pathway, which generates the farnesyl pyrophosphate substrate for FTase, is a central metabolic hub. Its inhibition by statins not only lowers cholesterol but also disrupts the farnesylation of key signaling proteins, contributing to their pleiotropic effects. For example, statins can induce apoptosis in cancer cells by depleting farnesyl pyrophosphate and inhibiting Ras farnesylation (Han and Wyche, 1996; Nakajima et al., 2007), and they can modulate inflammatory responses by affecting the farnesylation of proteins involved in NF-κB activation (Pahan et al., 1997). The delicate balance of protein prenylation is critical for tissue homeostasis, as demonstrated by the role of farnesylation in Sertoli cell function and fertility (Wang et al., 2013; Zhu et al., 2019), and in the progression of neurodegenerative diseases like Alzheimer’s, where FTIs have been shown to reduce amyloid pathology (Cuddy et al., 2022). The development of cardiomyocyte senescence and cardiac fibrosis has also been linked to defective lamin A maturation due to farnesyltransferase deficiency (Chen et al., 2026). Specifically, cardiomyocyte-specific deletion of the farnesyltransferase beta subunit (FNTB) in mice impairs lamin A maturation, destabilizes nuclear envelope integrity, and triggers a DNA damage response (DDR) characterized by increased Atm phosphorylation, elevated γH2AX foci formation, and Chk1/Chk2 activation, which drives cardiomyocytes into senescence rather than apoptosis (Chen et al., 2026). These senescent cardiomyocytes exhibit a senescence-associated secretory phenotype (SASP), secreting elevated levels of Tgf-β2 and Gdf15, which in turn activate cardiac fibroblasts and promote progressive interstitial fibrosis and diastolic dysfunction (Chen et al., 2026). Importantly, lipid overload suppresses FNTB transcription via Srebf2 downregulation, a mechanism recapitulated in hyperlipidemic human hearts showing reduced FNTB expression, and AAV9-mediated Fntb overexpression attenuates cardiac fibrosis in mice fed a high-fat diet, establishing a direct link between dysregulated farnesylation and lipotoxic cardiomyopathy (Chen et al., 2026). Thus, the nexus of lamin A biogenesis, farnesylation, and aging represents a fundamental biological axis with profound implications for understanding and treating a spectrum of human diseases, from rare progeroid syndromes to common age-related pathologies.

Our read of the evidence is that lonafarnib monotherapy will remain the proven standard of care for HGPS for the foreseeable future; the principal open questions are whether statin/bisphosphonate combination therapy, RCE1 modulation, and NAT10 inhibition can deliver additional benefit in head-to-head trials, and whether the same farnesyl-prelamin-A axis is druggable in sporadic vascular ageing, where the public-health stakes are far larger than in HGPS itself.

### Farnesylation in cell division and cytoskeletal dynamics

Farnesylation, the addition of a 15-carbon farnesyl isoprenoid lipid to C-terminal CAAX motifs, is a critical regulator of cell division and cytoskeletal organization. Its pharmacological inhibition profoundly disrupts mitosis and meiosis. Beyond these roles, farnesylation governs subcellular protein targeting; for example, PEX19 farnesylation dictates differential targeting of client proteins between the endoplasmic reticulum (ER), lipid droplets (LDs), and peroxisomes (Schrul and Kopito, 2016). The mevalonate pathway enzyme geranylgeranyl diphosphate synthase (GGPPS) is required for maintaining the blood–testis barrier (BTB). Sertoli cell-specific Ggpps deletion disrupts RhoA and Ras family protein isoprenylation, leading to BTB breakdown, loss of Sertoli cell–germ cell adhesions, hypospermatogenesis, and infertility (Zhu et al., 2019).

Farnesylation targets proteins to the kinetochore. The mitotic kinesin CENP-E requires C-terminal farnesylation for fibrous corona expansion, independent of its motor activity (Wu et al., 2024). Kinetochore targeting of the dynein adaptor Spindly is absolutely dependent on its farnesylation, which mediates interaction with the ROD-Zwilch-ZW10 (RZZ) complex, dynein-dynactin recruitment, and spindle assembly checkpoint (SAC) silencing (Moudgil et al., 2015; Mosalaganti et al., 2017). This RZZ-Spindly interaction drives fibrous corona oligomerization, potentiated by MPS1 kinase activity (Sacristan et al., 2018). Centromere protein F (CENP-F) farnesylation is essential for its kinetochore localization and critical for oocyte meiotic progression and female fertility (Zhong et al., 2026).

FTase inhibitors (FTIs) cause centrosome separation defects and donut-shaped nuclei via lamin B1 and pericentrin degradation, leading to aneuploidy and binucleation (Verstraeten et al., 2011). FTIs synergistically enhance mitotic arrest and cell death induced by microtubule-stabilizing agents, an effect linked to increased tubulin acetylation (Moasser et al., 1998; Marcus et al., 2005).

Farnesylation is essential for meiotic progression. Decreased protein farnesylation in aged granulosa cells compromises cumulus expansion and oocyte maturation. FTI-277 treatment recapitulates this aging phenotype (Zhang et al., 2026). Proteomic screening identified prostaglandin E2 synthase 2 (PTGES2) as a farnesylated protein; age-related decline in PTGES2 farnesylation reduces ER localization and PGE2 production, impairing cumulus expansion. Farnesol supplementation ameliorates these deficits in aged oocytes and mice (Zhang et al., 2026). CENP-F farnesylation is also required for oocyte meiosis (Zhong et al., 2026). In male reproduction, reduced GGPPS in non-obstructive azoospermia patients correlates with BTB disruption, a phenotype recapitulated by Ggpps deletion in mouse Sertoli cells due to aberrant RhoA and Ras isoprenylation (Zhu et al., 2019).

Farnesylation acts as a molecular switch for organelle-specific protein sorting. Farnesylated PEX19 prevents mistargeting of client proteins like UBXD8 to peroxisomes, instead promoting delivery to ER subdomains and LDs. A farnesylation-deficient PEX19 mutant causes UBXD8 mislocalization to peroxisomes (Schrul and Kopito, 2016). Similarly, PTGES2 farnesylation governs ER localization for PGE2 synthesis (Zhang et al., 2026), and geranylgeranylation-dependent membrane association of RhoA and Cdc42 maintains Sertoli cell junctions critical for spermatogenesis (Zhu et al., 2019).

### Farnesyltransferase inhibitors: development, mechanisms, and clinical applications

### Farnesyltransferase inhibitors: from bench to clinic

Building on the enzymology and substrate-recognition principles outlined earlier, FTIs were developed initially as broad-spectrum Ras-targeted anticancer agents and subsequently repurposed for HGPS and, most recently, for biomarker-selected HRAS-mutant tumours. The medicinal-chemistry arc—from CaaX tetrapeptide competitive inhibitors and benzodiazepine peptidomimetics (BZA-5B) (James et al., 1993) to the orally bioavailable non-peptidomimetics tipifarnib (R115777), lonafarnib (SCH66336), and BMS-214662, and onward to dual FTase/GGTase-I inhibitors such as L-778,123 and FGTI-2734 designed to overcome alternative prenylation—is summarised comprehensively in Table 10 and is not re-narrated here. Instead we focus on integrative points that span the boundaries between medicinal chemistry, pharmacology, and clinical result.

**TABLE 10:** Key farnesyltransferase inhibitors: development and properties.

Name	Content/Function	Citation (PMID)
BZA-5B	Benzodiazepine-based peptidomimetic FTI; IC50 < 1 nM; restored normal growth in Ras-transformed cells	James et al. (1993)
L-731,734	Prodrug; selectively inhibited Ras-dependent transformation	Kohl et al. (1993)
FTI-276	Selectively blocked growth of K-Ras-mutant human lung carcinoma in nude mice; correlated with inhibited Ras processing	Sun et al. (1995)
FTI-277	Radiosensitized H-ras-transformed fibroblasts	Bernhard et al. (1996)
L-744,832	Inhibited >70% of human tumor cell lines irrespective of ras status	Sepp-Lorenzino et al. (1995)
B956	Active against wild-type ras lines	Nagasu et al. (1995)
R115777 (tipifarnib)	Orally bioavailable non-peptidomimetic FTI; showed oral antitumor effects, particularly in H-ras or N-ras mutant lines	End et al. (2001)
SCH66336 (lonafarnib)	Orally bioavailable non-peptidomimetic FTI; suppressed tumor growth in xenograft and transgenic models	Nielsen et al. (1999)
BMS-214662	Orally bioavailable non-peptidomimetic FTI; exhibited broad-spectrum cytotoxicity	Ryan et al. (2004)
A-176120	FPP-competitive inhibitor	Tahir et al. (2000)
L-778,123	Dual prenylation inhibitor targeting FTase and GGTase-I to overcome K-Ras alternative prenylation	Britten et al. (2001), Lobell et al. (2001)

#### Pharmacodynamic biomarkers

Two biomarkers anchor the FTI pharmacology literature: the chaperone HDJ-2 (DNAJA1), whose unfarnesylated form accumulates dose-dependently with FTase inhibition (Gilardi et al., 2020; Cohen et al., 2003; Zimmerman et al., 2004), and prelamin A, which accumulates when its farnesylated form cannot be processed onward by ZMPSTE24 (Sinensky et al., 1994; Adjei et al., 2000). Standardised *in vitro* and cell-based prenylation assays built around [^3^H]-FPP/[^3^H]-GGPP and electrophoretic mobility shift (Berndt and Sebti, 2011), together with a fluorescent probe for real-time FTase imaging (Pham et al., 2006), enable cross-trial comparisons of target engagement and remain the methodological backbone for new FTI programmes.

#### The alternative-prenylation barrier

The single most important pharmacological fact about FTIs is that K-Ras and N-Ras can be geranylgeranylated by GGTase-I when FTase is inhibited (James et al., 1996; Lobell et al., 2001), rescuing their membrane association and oncogenic output. Conditional knockout of Fntb or Pggt1b in KRAS-G12D-driven models reduces tumorigenesis and extends lifespan (Berndt et al., 2011), validating the pathway but also confirming that monotherapy with an FTase-selective agent is unlikely to work in KRAS-driven solid tumours. This barrier is the mechanistic motivation for dual FTase/GGTase-I inhibitors (L-778,123; FGTI-2734) and for combination strategies with statins and aminobisphosphonates that deplete upstream isoprenoid pools (Varela et al., 2008; Gordon et al., 2016). It is also the reason the field pivoted toward indications in which the relevant substrate cannot fall back on geranylgeranylation—most notably HGPS (where progerin is the target) and HRAS-mutant tumours.

#### Single-agent clinical activity

The early Phase II programmes of tipifarnib in NSCLC, pancreatic adenocarcinoma, breast cancer, and SCLC (Adjei et al., 2003b; Cohen et al., 2003; Johnston et al., 2003; Heymach et al., 2004), and of lonafarnib in MDS/CMML (Feldman et al., 2008), are summarised in Table 7 and not re-listed here. The pattern is consistent: meaningful single-agent activity in haematologic malignancies (tipifarnib in refractory AML and CMML; lonafarnib in MDS/CMML) and negligible single-agent activity in unselected solid tumours. The HGPS programme—culminating in the FDA approval of lonafarnib (Zokinvy) on the basis of improved cardiovascular, bone, and survival outcomes (Gordon et al., 2014; Gordon et al., 2016; Misteli, 2021), and in a plasma-progerin pharmacodynamic assay (Gordon et al., 2023)— is covered in the Nuclear Lamins, Progeria, and the Farnesylation–Aging Nexus section above and is not re-described here.

#### HRAS-mutant tumours: the genotype-matched revival

The most consequential clinical development for this drug class has come from a genetically defined repositioning of tipifarnib in HRAS-mutant tumours. Unlike K-Ras and N-Ras, which carry a C-terminal polybasic stretch that allows GGTase-I-dependent geranylgeranylation to substitute for farnesylation when FTase is inhibited, H-Ras lacks this backup prenylation route and is therefore uniquely dependent on FTase. Gilardi and colleagues showed preclinically that FTase inhibition selectively mislocalises and disables HRAS oncogenic signalling, providing the mechanistic rationale for tumour-type selection by HRAS mutation status (Gilardi et al., 2020) This rationale was vindicated by the phase II AIM-HN trial of tipifarnib in HRAS-mutant head-and-neck squamous cell carcinoma (HNSCC), in which objective responses were observed in approximately half of the patients with high HRAS mutant-allele frequency, alongside a manageable safety profile dominated by myelosuppression and fatigue (Ho et al., 2021). Crucially, response was strongly modulated by HRAS mutant-allele frequency, identifying this as the first validated predictive biomarker for an FTI and converting tipifarnib from a failed broad-spectrum Ras-targeted agent into a genotype-matched therapy. In our view, this HRAS-mutant HNSCC programme is the most concrete clinical beachhead for the entire FTI class, and the principal open question is whether biomarker-selected indications beyond HRAS—for example, the INPP5A-defined NRAS/GNAQ/GNA11-mutant melanoma suggested by recent chemical proteomics (Pan et al., 2025)— can be developed on the same logic.

#### Combination strategies and FTI-sensitive pathways

Beyond the alternative-prenylation logic, several integrative pharmacology themes cut across the FTI literature. (i) Microtubule crosstalk. FTIs synergise with taxanes and epothilones by a Ras-independent mechanism: FTase physically associates with HDAC6 at microtubules, and FTase inhibition releases HDAC6, increases tubulin acetylation, and enhances mitotic arrest (Moasser et al., 1998; Marcus et al., 2005; Berndt et al., 2011). (ii) AIMP2-DX2/KRAS interface. The splicing variant AIMP2-DX2 stabilises pre-farnesylated KRAS by competitively blocking Smurf2-mediated ubiquitination (Kim et al., 2022), defining an indirect but farnesylation-aware therapeutic entry point. Small molecules that disrupt the AIMP2-DX2–KRAS interface reduce KRAS protein levels and suppress KRAS-dependent growth *in vitro* and *in vivo* (Kim et al., 2022). PROTAC degraders of AIMP2-DX2 have since been developed and shown to deplete DX2 in lung cancer cells (Lee et al., 2023), providing a candidate strategy—yet to be directly validated—for downregulating KRAS through DX2 elimination. (iv) PAK-mediated pro-survival bypass. Yeast chemical profiling and mammalian cell screens identified group I PAKs (CLA4/PAK1–3) as mediators of FTI resistance, with FTIs paradoxically inducing nuclear PhoPAK clustering; PAK inactivation potentiates FTI anti-proliferative action and identifies a rational combination partner (Porcu et al., 2013). (v) Neurodegeneration. Both LNK-754 and, less potently, lonafarnib enhance retrograde axonal transport of endolysosomes, stabilise microtubules, and reduce amyloid pathology in 5XFAD mice (Cuddy et al., 2022), extending FTI pharmacology into neurodegenerative disease.

#### Evaluative synthesis

Taken together, the clinical history of FTIs is best read as a story of indication selection: broad-spectrum Ras-targeted use failed because K-/N-Ras escape via alternative prenylation, but the same pharmacology is clinically active in HGPS, in unselected haematologic malignancies, and—most importantly—in biomarker-selected HRAS-mutant HNSCC. The most promising directions are therefore genotype-matched FTI use, dual prenylation blockade in KRAS-driven tumours, and chemical-proteomic nomination of new substrate-defined indications; the principal uncertainty is whether predictive biomarkers beyond HRAS mutant-allele frequency can be robustly developed.

## Emerging concepts and future directions

Recent work has established that K-Ras organises its signalling through farnesylation-dependent liquid–liquid phase separation (LLPS). The farnesyl moiety attached at Cys185 is the obligate biophysical driver of K-Ras condensate formation in the cytoplasm: removing or masking the farnesyl group abolishes condensation, and engineered analogues that restore hydrophobic lipidation restore droplet formation (Wang et al., 2026). These cytoplasmic condensates in turn compartmentalise the CaaX-processing machinery: RCE1 is recruited into K-Ras condensates, and this clustering enhances the endoproteolytic maturation of newly synthesised K-Ras, generating a self-reinforcing processing and signalling compartment (Wang et al., 2026). Pathologically, condensate burden correlates with colon-cancer stage, and high-condensate tumours show altered sensitivity to KRAS-G12C covalent inhibitors, with condensates acting as concentrating compartments that modulate drug engagement and effector output (Wang et al., 2026). In our read, LLPS is the most consequential conceptual advance in the Ras–farnesylation field of the past decade, because it converts farnesylation from a passive membrane-anchor function into an active, reaction-compartment organising principle—and it immediately raises the therapeutic question of whether condensate-modulating interventions can re-sensitise KRAS-mutant tumours to existing G12C-directed therapy.

Farnesylation allosterically modulates protein–protein interactions, as shown by PEX19, where the buried farnesyl group reshapes its surface to recognize peroxisomal membrane proteins (Emmanouilidis et al., 2017). Crosstalk with phosphorylation is exemplified by Rnd3–14-3-3 interactions, where distinct modification patterns tune binding thermodynamics (Hu et al., 2021), and by a farnesyl-electrostatic switch on K-Ras, where PKC phosphorylation at S181 promotes mitochondrial translocation and Bcl-XL–mediated apoptosis (Bivona et al., 2006). Farnesylation also primes palmitoylation, as seen with Ykt6 for intra-Golgi transport (Fukasawa et al., 2004), and Rho GTPase localization is governed by interplay between prenylation, palmitoylation, and RhoGDI binding (Michaelson et al., 2001). Cholesterol synthesis regulates Ras farnesylation and membrane association (Gadbut et al., 1997), while AIMP2-DX2 competitively blocks Smurf2-mediated ubiquitination of pre-farnesylated KRAS, stabilizing it to promote tumorigenesis (Kim et al., 2022).

Farnesylated prelamin A accumulation at the nuclear envelope triggers RIPK1-dependent necroptosis via RIPK3-mediated MLKL phosphorylation and nuclear disruption (Yang et al., 2024), representing a novel cell death mechanism relevant to progeroid syndromes.

Therapeutic strategies include dual FTase/GGTase inhibitors like FGTI-2734, which blocks KRAS membrane localization and inhibits mutant KRAS-driven pancreatic tumors (Kazi et al., 2019), and bivalent inhibitors disrupting protein surface–substrate interactions (Machida et al., 2011). Small molecules that disrupt the AIMP2-DX2–KRAS interface reduce KRAS protein levels and suppress KRAS-dependent growth (Kim et al., 2022). PROTAC degraders of AIMP2-DX2 have since been developed (Lee et al., 2023) and represent a candidate strategy to achieve KRAS depletion through DX2 elimination—a hypothesis yet to be directly tested in that study. Statins deplete prenyl pyrophosphate pools, inducing apoptosis in cancer cells (Kotamraju et al., 2007; Ogunwobi and Beales, 2008), inhibiting T-cell cytokine production (Marks et al., 2007), and differentially regulating monocyte/macrophage cytokines via Rac1 geranylgeranylation (Fu et al., 2019). Statins with aminobisphosphonates extend longevity in progeria models by blocking progerin prenylation (Varela et al., 2008), while farnesol enhances PARIS farnesylation to prevent muscle weakness in aged mice (Bae et al., 2023).

Key unanswered questions include defining the full farnesylated proteome via chemical proteomics (Charron et al., 2013; Storck et al., 2019), elucidating mechanisms of farnesylation-driven LLPS and PTM integration, understanding FTase/GGTase substrate specificity *in vivo*, and translating mechanistic insights into isoform-specific inhibitors, PROTACs, and strategies to fine-tune mevalonate pathway flux without systemic toxicity.

Over the next decade, this field is expected to be reshaped by two developments: the recognition that farnesylation drives LLPS-dependent signalling compartments rather than merely serving as a membrane anchor, and the maturation of quantitative chemical proteomics to a point where the farnesylated proteome is no longer the rate-limiting unknown. A common therapeutic horizon is pointed toward—the transition of FTIs from empirically selected to mechanistically and proteomically matched indications.

## Conclusion

In conclusion, protein farnesylation is a critical post-translational modification that governs the localization, interactions, and functions of a diverse array of proteins, thereby playing multifaceted roles in both health and disease. The covalent attachment of a farnesyl isoprenoid group, catalyzed by farnesyltransferase (FTase), is essential for the biological activity of key regulatory proteins, including the Ras superfamily of small GTPases, nuclear lamins, and various signaling molecules (Casey et al., 1989; Manne et al., 1990; Reiss et al., 1990). This modification is not merely a membrane anchor; it can allosterically regulate protein conformation, as demonstrated by the farnesyl-dependent autoinhibition of the SNARE protein Ykt6 (Wen et al., 2010), and can serve as a critical determinant for protein-protein interactions, such as the recognition of farnesylated peptides by PDEδ (Dharmaiah et al., 2016). The fundamental importance of this pathway is underscored by its conservation across eukaryotes, from yeast and plants to humans, where it regulates processes ranging from cell cycle progression and meristem development to abiotic stress responses (Goodman et al., 1990; Qian et al., 1996; Yalovsky et al., 2000a; Barghetti et al., 2017; Feng et al., 2021).

The central role of farnesylation in oncogenic Ras signaling provided a strong rationale for the development of farnesyltransferase inhibitors (FTIs) as anticancer agents. Preclinical studies demonstrated that FTIs could potently and selectively block the farnesylation of H-Ras, reverse the transformed phenotype of H-Ras-driven tumors, and inhibit tumor growth in xenograft models (James et al., 1993; Kohl et al., 1993; Nagasu et al., 1995; Sun et al., 1995). These findings were extended to other Ras-dependent malignancies, including neurofibromatosis type 1 and Bcr/Abl-positive leukemias, where FTIs showed significant promise (Yan et al., 1995; Reichert et al., 2001). The therapeutic successes of FTIs, however, have been more nuanced in the clinical setting. While some hematologic malignancies, such as acute myelogenous leukemia and chronic myelomonocytic leukemia, have shown objective responses to FTIs like tipifarnib and lonafarnib, their efficacy in solid tumors harboring the more prevalent K-Ras mutations has been limited (Adjei et al., 2003b; Cohen et al., 2003; Lancet et al., 2007; Feldman et al., 2008). A major mechanism of resistance was uncovered when it was found that K-Ras and N-Ras can undergo alternative prenylation by geranylgeranyltransferase I (GGTase-I) upon FTase inhibition, thereby maintaining their membrane localization and transforming activity (James et al., 1996; Lobell et al., 2001). This critical insight has shifted the therapeutic paradigm from selective FTase inhibition to strategies that target both branches of the prenylation pathway.

The limitations of FTIs have spurred the development of next-generation prenylation-targeted therapies with broader applications. Dual farnesyltransferase and geranylgeranyltransferase inhibitors (DPIs) have been designed to overcome the alternative prenylation resistance mechanism and have shown enhanced antitumor activity in preclinical models of K-Ras-driven cancers (Lobell et al., 2001; Liu et al., 2010; Kazi et al., 2019). Beyond oncology, the therapeutic targeting of protein farnesylation has found remarkable success in treating the premature aging disorder Hutchinson-Gilford progeria syndrome (HGPS). The disease is caused by a mutant form of prelamin A, progerin, which retains a farnesyl group that is not cleaved, leading to its toxic accumulation at the nuclear envelope and severe nuclear blebbing (Capell et al., 2005; Mallampalli et al., 2005). FTIs have been shown to reverse these cellular phenotypes and, in clinical trials, the FTI lonafarnib (Zokinvy) has improved cardiovascular and bone disease and significantly extended survival in children with HGPS, leading to its FDA approval (Toth et al., 2005; Yang et al., 2005; Gordon et al., 2014; Misteli, 2021). Combination therapies that further inhibit progerin prenylation, such as lonafarnib with pravastatin and zoledronic acid, are being explored to enhance clinical benefit (Varela et al., 2008; Gordon et al., 2016).

The therapeutic promise of targeting farnesylation is expanding into neurodegenerative and infectious diseases. In Parkinson’s disease, farnesylation of the parkin-interacting substrate PARIS promotes its neurotoxicity, and farnesyltransferase inhibitors or the natural compound farnesol can prevent neurodegeneration in animal models (Jo et al., 2021; Bae et al., 2023). Similarly, farnesylation of UCH-L1 promotes α-synuclein neurotoxicity, and FTIs have shown protective effects in cellular models (Liu et al., 2009). In Alzheimer’s disease models, the FTI LNK-754 has been shown to attenuate axonal dystrophy and reduce amyloid pathology (Cuddy et al., 2022). The host farnesylation machinery is also exploited by intracellular pathogens. *Legionella pneumophila* requires farnesylation of its effector protein AnkB for anchoring to the pathogen-containing vacuole, a process essential for bacterial proliferation (Price et al., 2010; 2011). Inhibiting this host-mediated modification represents a novel antimicrobial strategy. Furthermore, targeting farnesylation has shown potential in treating parasitic infections like Chagas disease, where inhibitors of Trypanosoma cruzi sterol 14-demethylase, initially identified in FTase screens, have potent anti-parasitic activity (Buckner et al., 2003). The continued development of advanced chemical tools, including photoaffinity and bioorthogonal probes for profiling prenylated proteins, will be instrumental in identifying new therapeutic targets and realizing the full potential of prenylation-targeted therapies across this wide spectrum of human diseases (Chehade et al., 2002; Kho et al., 2004; Storck et al., 2019).
